# Altered immunity in migraine: a comprehensive scoping review

**DOI:** 10.1186/s10194-024-01800-8

**Published:** 2024-06-07

**Authors:** Woo-Seok Ha, Min Kyung Chu

**Affiliations:** Department of Neurology, Severance Hospital, Yonsei University College of Medicine, 50-1, Yonsei-ro, Seodaemun-gu, Seoul, 03722 Republic of Korea

**Keywords:** Immunity, Lymphocytes, Migraine, Neuroinflammation, Autoimmune diseases, Allergy, Inflammation

## Abstract

**Background:**

The pathogenesis of migraine remains unclear; however, a large body of evidence supports the hypothesis that immunological mechanisms play a key role. Therefore, we aimed to review current studies on altered immunity in individuals with migraine during and outside attacks.

**Methods:**

We searched the PubMed database to investigate immunological changes in patients with migraine. We then added other relevant articles on altered immunity in migraine to our search.

**Results:**

Database screening identified 1,102 articles, of which 41 were selected. We added another 104 relevant articles. We found studies reporting elevated interictal levels of some proinflammatory cytokines, including IL-6 and TNF-α. Anti-inflammatory cytokines showed various findings, such as increased TGF-β and decreased IL-10. Other changes in humoral immunity included increased levels of chemokines, adhesion molecules, and matrix metalloproteinases; activation of the complement system; and increased IgM and IgA. Changes in cellular immunity included an increase in T helper cells, decreased cytotoxic T cells, decreased regulatory T cells, and an increase in a subset of natural killer cells. A significant comorbidity of autoimmune and allergic diseases with migraine was observed.

**Conclusions:**

Our review summarizes the findings regarding altered humoral and cellular immunological findings in human migraine. We highlight the possible involvement of immunological mechanisms in the pathogenesis of migraine. However, further studies are needed to expand our knowledge of the exact role of immunological mechanisms in migraine pathogenesis.

**Supplementary Information:**

The online version contains supplementary material available at 10.1186/s10194-024-01800-8.

## Background

Neurogenic inflammation and neuroinflammation have been implicated to play a key role in migraine pathogenesis [1–4]. Defined as an acute, sterile inflammation, neurogenic inflammation occurs when nociceptive fibers release neural mediators, leading to vasodilation and plasma extravasation [5, 6]. Both neurogenic inflammation and neuroinflammation are mediated by immunological processes, including the action of cytokines and the involvement of immune cells [1]. Thus, a deeper understanding of the immunological alterations in migraine could shed light on the roles of neurogenic inflammation and neuroinflammation in its mechanism. This insight could also provide the groundwork for future research into the pathogenesis of migraine.

Preclinical models have demonstrated activation of the trigeminovascular system, which induces local neurogenic inflammation involving the meninges and dural and pial vessels [7].

Nonsteroidal anti-inflammatory drugs reduce inflammation and have been used effectively to treat migraine attacks. Calcitonin gene-related peptide (CGRP) plays a pivotal role in migraine pathogenesis and is involved in host immune surveillance and immunomodulatory activities [8, 9]. Furthermore, significant changes in markers related to immunity have been observed during the ictal and interictal periods [10, 11]. In this review, we aimed to scope the changes of immune-related markers in migraine.

## Methods

### Search strategies and selection of articles

To identify articles on immunological changes in human migraine, a systematic electronic search was conducted using the PubMed database. This search followed the Preferred Reporting Items for Systematic Reviews and Meta-Analyses guidelines [12]. The search was carried out on 23 December 2023, using the combination of the keywords “migraine AND (immune OR immunity OR cytokine OR lymphocyte)”. We entered these search terms as free text in PubMed and set no limits to avoid excluding pertinent records.

### Inclusion criteria for articles

Our selection included original cohort studies, clinical trials, reviews, or systematic analyses. To qualify for inclusion, papers had to meet the following criteria: published up to December 2023, written in English, involve more than 20 human subjects with migraine, and measure or focus on specified immune substances of interest. To capture the diverse dimensions in measuring immune changes, we included quantitative, qualitative, and mixed-method studies.

### Selection process

**Fig. 1:**
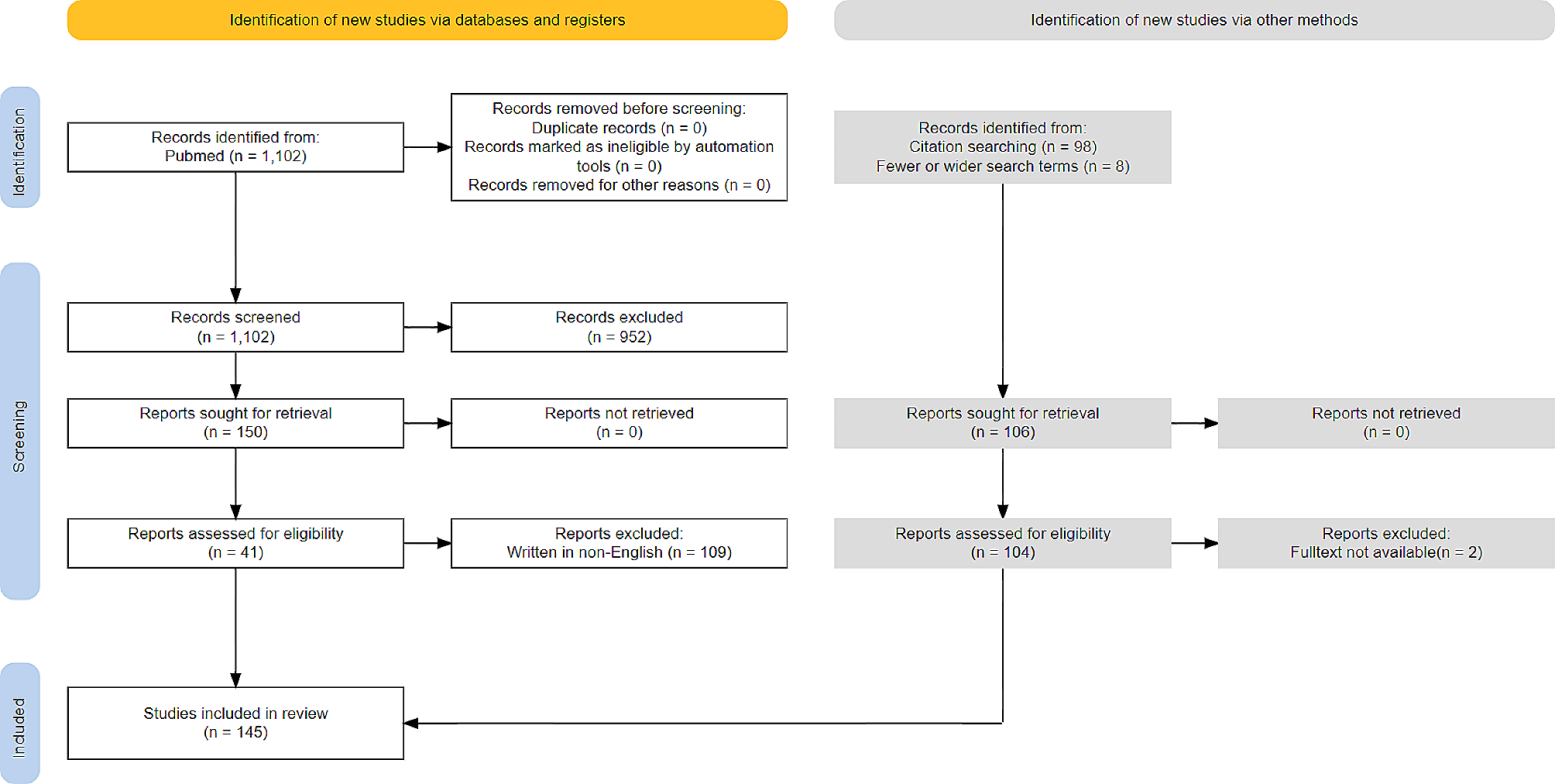
PRISMA literature search flowchart. PRISMA, preferred reporting items for systematic reviews and meta-analyses

The initial PubMed search yielded 1,102 records. Two authors (WS Ha and MK Chu) reviewed these records for relevance, ultimately selecting 41 for inclusion. Additional searches were performed using the reference lists of the included studies, or fewer or wider search terms, after which an additional 104 articles were included for review. The eligibility of further relevant articles was assessed through full-text screening independently by both authors, with the final selection being made based on a consensus between the two authors. In total, 145 articles were reviewed for this study (Fig. 1) [13].

### Data collection and analysis

We recorded qualitative outcomes from the studies, focusing on changes with statistical significance, in Excel tables. Two authors independently extracted data into this table from the pre-screened lists. Disagreements were resolved through discussion, and the data-charting form was iteratively updated to ensure comprehensive accuracy. If episodic migraine (EM) and chronic migraine (CM) were described separately, we referred to them as EM and CM, respectively. If EM and CM were described separately in the studies, they were described separately; if not, they were described as migraine. Risk of bias was assessed using the Newcastle–Ottawa Quality Assessment Scale [14].

For each study, we collected qualitative changes in immune-related markers during the interictal or ictal period. Changes were categorized as increase, no change, or decrease. Statistical significance was defined as two-tailed *p* < 0.05, unless otherwise specified. When papers specified ictal or interictal periods for sampling, we adhered to those indications; otherwise, we assumed an interictal period. The change during the interictal period was defined as a comparison of the measurements in the patients with migraine with those in controls. The change during the ictal period was defined as a comparison of the measurements in the ictal period and the interictal period within the patients with migraine. In addition, we collected quantitative changes in immune-related markers by group if the study specified these measurements. The measurement data included means and standard deviation. If only measurements for subgroups were reported, we calculated the mean and standard deviation for the combined group. If only the standard error of the mean was reported, we calculated the standard deviation based on the number of samples [15]. Random effects meta-analyses were performed for measures with five or more studies, using the software RevMan 5 (Cochrane Collaboration, London, United Kingdom).

## Results

### Humoral immunity

#### Cytokines

##### IL-1β

IL-1β is an important mediator of the human body’s response to infection, inflammation, and immunological conditions. Twelve studies measured interictal blood IL-1β levels, with seven reporting an increase in the migraine group compared to that in controls [16–22], and five studies reporting no significant difference [23–27]. Nine studies evaluated ictal blood levels of IL-1β and showed mixed results: four studies reported elevated levels [18, 21, 26, 28], and five studies reported no change [20, 23, 24, 27, 29] (Table 1). The meta-analysis including five studies on IL-1β levels at interictal periods found no significant difference compared to that in controls, while the analysis including five studies with IL-1β levels at ictal periods found significant increases compared to those in interictal periods (Supplementary Material S1).

##### IL-6

IL-6 is a major proinflammatory cytokine produced at the site of inflammation and tissue damage by macrophages, T and B lymphocytes, eosinophils, basophils, and various immune cells. Nineteen studies investigated the interictal levels of IL-6 in individuals with migraine, with mixed results: 11 studies reported increased levels [17–21, 27, 30–34], six reported no change [16, 22, 24, 26, 35, 36], and two showed decreased levels [23, 25]. Ictal levels were reported in 11 studies, with increased levels in three [18, 21, 27], no change in seven [20, 23, 26, 28, 30, 34, 36], and decreased levels in one study [37]. The meta-analysis including 14 studies on IL-6 levels at interictal periods found significantly increased levels compared to controls, while the analysis including nine studies at ictal periods found no significant differences compared to those in interictal periods (Supplementary Material S2).

##### TNF-α

TNF-α is a proinflammatory cytokine with a primary role in the regulation of inflammation and is involved in coagulation, lipid metabolism, and apoptosis. Twenty studies investigated interictal blood levels of TNF-α and revealed inconsistent results: levels were increased in nine studies [17–19, 22, 25, 30, 38], there was no significant change in 10 studies [16, 20, 23, 24, 27, 31, 35, 36, 39, 40], and TNF-α levels decreased in one study [26]. Twelve studies investigated ictal changes and found mixed results: an increase in three studies [18, 21, 26] and no change in nine studies [20, 23, 27–30, 36, 40, 41]. The meta-analysis including 12 studies on TNF-α levels at interictal periods found significantly increased levels compared to those in controls, while the analysis including seven studies at ictal periods found marginally increased levels compared to those in interictal periods (Supplementary Material S3).

##### IL-4

IL-4 is an anti-inflammatory cytokine that induces the proliferation and differentiation of T and B lymphocytes, regulates the functions of mast cells and macrophages, and inhibits the production of inflammatory cytokines. Five studies measured IL-4 levels during the interictal period: levels increased in two studies [22, 42], were unchanged in two [26, 30], and decreased in one [19]. Five studies reported ictal levels, with three reporting decreased levels [28, 37, 43] and two reporting no significant differences [26, 30].

##### IL-10

IL-10 is an anti-inflammatory cytokine that inhibits the synthesis of proinflammatory cytokines and terminates the inflammatory response. Eleven studies evaluated interictal IL-10 levels, which were increased in two studies [16, 22], unchanged in four studies [26, 27, 30, 42], and decreased in five studies [19, 20, 25, 31, 35]. Four studies measured ictal levels: two reported elevated levels [26, 27], whereas another two reported no changes [20, 30]. The meta-analysis including nine studies on interictal levels of IL-10 showed no significant difference compared to those in controls (Supplementary Material S4).

##### TGF-β

TGF-β is a multifunctional cytokine that controls cell proliferation, motility, differentiation, and apoptosis. Cytokines also play a role in blood vessel formation and immune regulation. Two studies evaluated TGF-β levels during the interictal period and found elevated levels [44, 45]. No study reported ictal levels of TGF-β.

##### Other cytokines

IL-2, IL-8, IL-12, and IFN-γ are proinflammatory cytokines whose levels have been measured in the blood of individuals with migraine. Ictal and interictal IL-2 and IFN-γ levels have shown conflicting results [19, 20, 26, 27, 30, 35, 37, 42, 46]. Interictal IL-8 levels were investigated in six studies with mixed results [19, 22, 25, 28, 35, 47], and interictal IL-12 levels were measured in one study, which reported increased levels thereof [25].

#### Chemokines

Chemokines and chemotactic cytokines are a family of small cytokines, or signaling proteins, secreted by cells that induce the directional movement of leukocytes. Interictal blood levels of chemokine ligands (CCL) 2, CCL3, CCL4, and CCL5 were elevated in three, three, one, and two studies, respectively, while the levels were reported as no changes in two, one, one, and three studies, respectively [22, 27, 47–49].

#### Adipokines

Adipokines are cytokines secreted by adipose tissue and play a role in immune regulation [50]. Altered levels of adiponectin (ADP) and leptin have been reported in patients with migraine. ADP oligomerizes and circulates in the blood in high molecular weight (HMW), middle molecular weight (MMW), and low molecular weight (LMW) forms. HMW-ADP has proinflammatory functions, whereas LMW-ADP has anti-inflammatory functions. Total ADP (T-ADP) levels are reported to be elevated in patients with chronic daily headaches [51, 52]. The increase in T-ADP levels in patients with migraine is mostly due to an increase in HMW-ADP and MMW-ADP levels. After successful acute treatment, the T-ADP levels decreased. Among acute treatment responders, HMW-ADP levels decreased and LMW-ADP levels increased, whereas only LMW-ADP levels decreased in those who do not respond to acute treatment [53]. Six studies reported on the interictal levels of total ADP, of which only two showed a significant increase compared to the levels in controls [16, 51, 54–57]. Similarly, six studies reported on interictal levels of leptin, with one showing a decrease, but the remaining five showed no significant difference [16, 54–56, 58, 59]. The meta-analysis included six studies each measuring total ADP and leptin levels in the interictal period, none of which showed significant results (Supplementary Material S5).

**Table 1:** Studies on ictal and interictal blood cytokine levels in individuals with migraine

First author, year	Country	No. of patients with migraine (% female)	Migraine types	No. of controls (% female)	Matched factors	Blood sample	NOS	Interictal levels	Ictal levels
ArmaĞAn, 2020 [18]	Turkey	Ictal: 30 (100%) Interictal: 30 (100%)	NR	30 (100%)	NR	Plasma	3	IL-1β (↑), IL-6 (↑), TNF-α (↑)	IL-1β (↑), IL-6 (↑), TNF-α (↑)
Aydın, 2015 [30]	Turkey	Ictal: 35 (69%) Interictal: 35 (71%)	MA: 30 (73%), MO: 40 (68%)	19 (73%)	Age, sex	Plasma	6	IL-6 (↑), TNF-α (↑), INF-γ (=), IL-4 (=), IL-10 (=)	IL-6 (=), TNF-α (=), INF-γ (=), IL-4 (=), IL-10 (=)
Bernecker, 2011-1 [54]	Austria	50 (84%)	MA: 20	74 (62%)	NR	Serum	4	Total ADP (=), Leptin (=)	
Bernecker, 2011-2 [55]	Austria	48 (100%)	MA: 17 (100%)	48 (100%)	NR	Serum	4	Total ADP (=), Leptin (=)	
Boćkowski, 2009 [39]	Poland	21 (48%)	MA: 9 (44%), MO: 12 (50%)	ETTH: 24 (75%)	NR	Plasma	3	TNF-α (=)	
Chai, 2015 [53]	USA	34 (94%)	All EM	Patients	N/A	Serum	7		Total ADP (↑), Leptin (=)
Chaudhry, 2019 [16]	Germany	30 (97%)	EM: 19, CM: 11 MA: 11, MO: 19	18 (83%)	NR	Serum	5	IL-1β (↑), IL-6 (=), TNF-α (=), Total ADP (=), Leptin (=), IL-10 (↑)	
Cowan, 2021 [35]	USA	42	Interictal EM: 24 (79%) Ictal EM: 13 (77%) CM: 16 (94%)	14 (71%)	Age, sex, SES	Plasma	8	IL-6 (=), TNF-α (=), INF-γ (=), IL-10 (↓)	
Dearborn, 2014 [56]	USA	Migraine: 72 (75%) Probable migraine: 59 (70%)	NR	850 (61%)	NR	Plasma	6	Total ADP (↑), Leptin (=)	
Domingues, 2016 [49]	Brazil	68 (93%)	NR	TTH: 48 (90%)	NR	Serum	4	CCL2 (↑), CCL3 (↑), CCL5 (↑)	
Domínguez, 2018 [31]	Spain	72 (83%)	All CM	24 (96%)	NR	Serum	6	IL-6 (↑), TNF-α (=), IL-10 (↓)	
Duarte, 2014 [57]	Brazil	68 (93%)	EM: 45, CM: 23 MA: 10	65 (88%)	NR	Serum	4	Total ADP (↑)	
Duarte, 2015 [47]	Brazil	49 (94%)	EM: 35, CM: 14 MA: 8	49 (94%)	Age, sex	Serum	6	CCL2 (↑), CCL3 (↑), CCL5 (=)	
Empl, 2003 [36]	Germany	Ictal: 6 (100%) Interictal: 21 (90%)	MA: 19 (100%), MO: 8 (75%)	8 (75%)	NR	Serum	4	IL-6 (=), TNF-α (=)	IL-6 (=), TNF-α (=)
Fidan, 2006 [27]	Turkey	Ictal: 25 (80%) Interitcal: 25 (80%)	MA: 7, MO: 18	25 (80%)	Age, sex	Serum	6	IL-1β (=), IL-2 (=), IL-6 (↑), TNF-α (=), INF-γ (=), CCL2 (=), CCL3 (=), CCL4 (=), CCL5 (=), IL-10 (=)	IL-1β (=), IL-2 (=), IL-6 (↑), TNF-α (=), INF-γ (=), CCL2 (=), CCL3 (=), CCL4 (=), CCL5 (↑), IL-10 (↑)
Flook, 2019 [22]	Spain	82 (65%)	All VM MA: 48, MO: 34	66	Serum	NR	3	IL-1β (↑), IL-6 (=), TNF-α (↑), INF-γ (↑), CCL2 (↑), CCL3 (↑), CCL4 (↑), CCL5 (↑), IL-4 (↑), IL-10 (↑)	
Guldiken, 2008 [58]	Turkey	61 (77%)	All EM	64 (66%)	Age, sex	Serum	5	Leptin (↓)	
Guo, 2017 [40]	Denmark	32 (81%)	All MO	6 (67%)	Age, sex	Serum	8	TNF-α (=)	TNF-α (=)
Güzel, 2013 [45]	Turkey	51 (71%)	MA: 27 (74%), MO: 24 (67%)	27 (59%)	NR	Serum	4	TGF-β (↑)	
Hirfanoglu, 2009 [17]	Turkey	77 (57%)	MA: 9, MO: 68	19	Serum	NR	5	IL-1β (↑), IL-6 (↑), TNF-α (↑)	
Ishizaki, 2005 [44]	Japan	68 (78%)	MA: 23 (65%), MO: 45 (84%)	59 (59%)	NR	Plasma	4	TGF-β (↑)	
Karaaslan, 2020 [23]	Turkey	30 (90%) Ictal: 11, Interictal: 19	All VM MA: 9	50 (70%)	Age, sex	Serum	5	IL-1β (=), IL-6 (↓), TNF-α (=)	IL-1β (=), IL-6 (=), TNF-α (=)
Koçer, 2009 [32]	Turkey	66 (91%)	All CM	45	Age, sex	Serum	6	IL-6 (↑)	
Lee, 2015 [19]	Taiwan	15 (73%)	All VM	15 (67%)	Age	Plasma	5	IL-1β (↑), IL-2 (↑), IL-6 (↑), TNF-α (↑), INF-γ (↑), IL-4 (↓), IL-10 (↓)	
Ligong, 2015 [59]	China	52 (67%)	NR	52 (67%)	Age, sex, BMI	NR	6	Leptin (=)	
Martami, 2018 [38]	Iran	43 (79%)	EM: 20, CM: 23	40 (78%)	Age, sex	Serum	5	TNF-α (↑)	
Martelletti, 1993 [37]	Italy	20 (80%)	NR	Patients	N/A	Serum	6		IL-6 (↓), INF-γ (↑), IL-4 (↓)
Martelletti, 1997 [43]	Italy	20 (65%)	All MO	20 (65%)	Age, sex	Serum	6		IL-4 (↓)
Mueller, 2001 [24]	USA	19 (100%)	All MRM	10 (100%)	NR	Serum	5	IL-1β (=), IL-6 (=), TNF-α (=)	IL-1β (=)
Munno, 1998 [42]	Italy	32 (72%)	All MO	32 (72%)	Age, sex	Plasma	5	INF-γ (=), IL-4 (↑), IL-10 (=)	
Oliveira, 2017 [25]	Brazil	20 (100%)	All EM	17 (100%)	Age, sex	Plasma	6	IL-1β (=), IL-6 (↓), IL-12 (↑), TNF-α (↑), IL-10 (↓)	
Perini, 2005 [26]	Italy	25 (88%) Ictal: 25, Interictal: 25	MA: 4, MO: 22	18 (72%)	NR	Plasma	6	IL-1β (=), IL-2 (↓), IL-6 (=), TNF-α (↓), IL-4 (=), IL-10 (=)	IL-1β (↑), IL-2 (=), IL-6 (=), TNF-α (↑), IL-4 (=), IL-10 (↑)
Peterlin, 2008 [51]	USA	25 (100%)	EM: 13 (100%) Chronic daily headache: 12 (100%)	12 (100%)	Age, sex, BMI	Serum	8	Total ADP (=)	
Sarchielli, 2004 [48]	Italy	8 (63%)	All MO, All EM	15 (60%)	Age	Serum	8	CCL2 (=), CCL5 (=)	CCL2 (=), CCL5 (=)
Sarchielli, 2006 [28]	Italy	7 (57%)	All MO, All EM	Patients	N/A	Plasma	8		IL-1β (↑), IL-6 (=), TNF-α (=), IL-4 (↓)
Shimomura, 1991 [46]	Japan	13 (62%)	All MO	43 (65%)	NR	Serum	5	IL-2 (↓)	
Tanure, 2010 [41]	Brazil	9 (89%)	All EM	Patients	N/A	Serum	5		TNF-α (=)
Togha, 2020 [33]	Iran	71 (86%)	EM: 44, CM: 27	19 (79%)	Age, sex	Serum	7	IL-6 (↑), TNF-α (↑)	
Uzar, 2011 [20]	Turkey	64 (70%) Ictal: 25, Interictal: 39	MA: 25, MO: 39	34 (71%)	NR	Serum	5	IL-1β (↑), IL-2 (=), IL-6 (↑), TNF-α (=), IL-10 (↓)	IL-1β (=), IL-2 (=), IL-6 (=), TNF-α (=), IL-10 (=)
van Hilten, 1991 [29]	Netherlands	20 (60%)	MA: 6, MO: 14	Patients	N/A	Plasma	6		IL-1β (=), TNF-α (=)
Wang, 2015 [34]	China	103 (41%)	MA: 31. MO: 72	100 (59%)	Age, sex	Serum	6	IL-6 (↑)	IL-6 (=)
Yücel, 2016 [21]	Turkey	31 (68%)	All EM	24 (63%)	NR	Serum	4	IL-1β (↑), IL-6 (↑), TNF-α (↑)	IL-1β (↑), IL-6 (↑), TNF-α (↑)

NOS: Newcastle–Ottawa Scale, NR: not reported, MA: migraine with aura, MO: migraine without aura, EM: episodic migraine, ETTH, episodic tension-type headache, SES: social economic status, CM: chronic migraine, TTH: tension-type headache, VM: vestibular migraine, N/A: not applicable, ↑: significantly increased, =: no significant difference, ↓: significantly decreased

#### Cell adhesion molecules and matrix metalloproteinases

Cell adhesion molecules (CAMs) are a subset of cell surface proteins involved in the binding of cells with other cells or other extracellular matrixes. CAMs participate in immune cell binding and are involved in immune regulation. The inhibition of intercellular adhesion molecule-1 (ICAM-1) causes decreased serum ICAM-1 levels during the ictal period [43].

Matrix metalloproteinases (MMPs) are a family of proteolytic enzymes capable of degrading extracellular matrix proteins and playing a role in immune regulation, including leukocyte recruitment, cytokine and chemokine processing, and matrix remodeling [60]. There are several subtypes of MMPs. Different results have been reported depending on the MMP subtype and the period of the migraine attack. Elevated overall MMP activity was observed in the migraine group compared to that in controls in one study [54], and decreased levels of MMP-3 were observed during the ictal period compared with those in the interictal period. The study found no significant differences in MMP-7, MMP-8, and MMP-10 levels between the ictal and interictal periods [61], and no significant differences between MMP-7 and MMP-8 levels. Interictal MMP-9 levels were increased in one study and unchanged in two other studies [54, 61, 62].

#### High-sensitivity C-reactive protein (CRP)

CRP is a circulating protein whose levels increase in response to inflammation. It originates in the liver after IL-6 secretion by macrophages and T lymphocytes. Since the traditional measurement of CRP has a measurable range of 10–1,000 mg/L, high sensitivity CRP (hsCRP) has been used to measure in the 0.5–10 mg/L range. Studies have reported hsCRP levels in patients with migraine. However, these results were contradictory; some showed elevated hsCRP levels in individuals with migraine compared to those in controls, while others showed no change. A review of eight positive and six negative studies raised the possibility that differences in CRP levels in migraine patients may depend on body mass index (BMI) distribution [63]. If the mean BMI of the patients was overweight or obese (≥ 25 kg/m^2^), the individuals in the migraine group had higher CRP levels than those in the control group. In contrast, if the mean BMI was less than that of overweight individuals (< 25 kg/m^2^), there was no difference between the migraine and normal groups [63]. Two studies examined CRP levels separately in migraine with aura (MA) and migraine without aura (MO), with one study showing a greater increase in migraine with aura than in migraine without aura and one study showing no difference [64, 65]. However, the association between migraine and CRP levels is inconclusive, with studies showing diverse outcomes.

#### Complement system

The complement system is a part of the immune system that enhances the ability of antibodies and phagocytic cells to clear microbes and damaged cells from an organism and promote inflammation. Significant decreases in C4 and C5 levels have been observed during migraine attacks, and significant decreases in C1s, C4, and factor-B have been reported in both MA and MO during migraine attacks [66]. These findings suggest complement system activation during migraine attacks [67, 68]. However, another study reported no changes in the complement system during migraine attacks [69]. The complement system in the interictal period has been reported in one study, which observed a decrease in C3 and no difference in C4 in the migraine group compared with that in the control group [70]. In summary, significant changes in some components of the complement system during the ictal and interictal periods have been observed in most studies.

#### Immunoglobulins

Three studies observed increases in IgM and IgA in patients with migraine compared with those in controls [66, 68, 71], whereas one study reported no difference [70]. Three studies assessed IgE levels; two found increased levels in migraine patients with allergic diseases, whereas one found decreased levels [72–74]. Two of these studies further compared IgE levels in migraine patients with and without allergies, with one study finding no difference and the other finding increased IgE levels in patients with allergies [73, 74].

#### Histamine

Histamine is a biogenic monoamine involved in local inflammation and acts as a key mediator of mast cells [75–77]. Four studies evaluated blood histamine levels in individuals with migraine during the interictal period; three studies reported increased levels compared to those in controls, and one study reported decreased levels [72, 73, 78, 79]. Histamine levels during the ictal period were observed in two studies, both of which showed a significant increase [78, 79].

#### Other immune-related blood markers

Altered blood levels of pentraxin 3 (PTX3) and cyclooxygenase-2 (COX-2) have been reported in migraine patients. PTXs are acute-phase proteins along with hsCRP, which are markers of endothelial dysfunction. Elevated levels of PTX3 during the ictal period have been reported [80–82]. During the interictal period, elevated PTX3 levels have been reported in patients with CM [83]. However, there have been no reports on interictal PTX3 levels in EM.

COX-2 is an enzyme involved in the conversion of arachidonic acid to prostaglandin H2, an important precursor of prostacyclin, which is expressed during inflammation. Elevated COX-2 levels during the ictal period have been reported in one study. Nevertheless, there was no significant difference in COX-2 levels between the migraine and control groups during the interictal period [84].

#### Cerebrospinal fluid (CSF) markers related to immunity

CSF is thought to reflect biochemical changes in the brain; therefore, it is the body fluid of primary interest in brain diseases [85]. Although neurogenic inflammation in the meninges and meningeal vessels in patients with migraine is likely to be local, immune-related marker changes may be detectable in the CSF. CSF levels of substances with high molecular weights are likely to reflect autologous production in the presence of an intact blood-brain barrier [86]. Most studies have been conducted during the interictal period because of the nature of CSF studies. Elevated CSF levels of immune-related markers in migraine are thought to reflect an inflammatory response in the nervous system; however, their exact significance remains unclear.

There are few studies on cytokine levels in the CSF. In a Norwegian hospital-based study, increased CSF levels of monocyte chemoattractant protein-1 and TGF-β were observed in the CSF of migraine patients. However, IL-1β, IL-4, and IL-10 were undetectable [87]. Another study in the USA found elevated levels of TNF-α in patients with treatment-refractory CM [88]. A further study in the USA found no significant differences in CSF levels of IL-6, IL-8, IL-10, hsCRP, and MMP-9 between individuals with migraine and controls [35]. CSF levels of CGRP have not been reported in EM, but increased levels have been reported in three studies on CM [89–91]. A meta-analysis has reported significantly elevated CSF CGRP levels in individuals with CM [92].

### Cellular immunity

Alterations in immune cell subsets have been observed in individuals with migraine [93]. A significant increase in CD4+ (T helper) and a decrease in CD8+ (cytotoxic T) lymphocytes have been observed in the blood during the interictal period [94, 95]. Blood tests in individuals with migraine revealed decreased monocyte counts and polymorphonuclear leukocyte (PMN) phagocytosis [96].

A flow cytometric study found an increase in the CD3 + CD16 + CD56 + lymphocyte subset, an NK cell subtype, in individuals with migraine during the ictal and interictal periods, compared to that in controls [97]. Another change in T lymphocyte subsets is a decrease in regulatory T (Treg) cells in individuals with migraine [98]. Considering that Treg cells play an important role in the prevention of autoimmunity, a decrease therein may lead to the failure of self-recognition and the development of autoimmune diseases. In summary, immune cell subset changes toward a proinflammatory pattern were observed, similar to those observed for cytokines.

### Genetic findings related to immunity in migraine

Genetic studies of migraine have shown differences in genes related to immunity. The immunogenetic changes identified in migraine include histocompatibility and cytokine gene polymorphisms.

#### Human lymphocyte antigen (HLA) gene polymorphism

HLA codes for major human histocompatibility complex (MHC) class I and II molecules. HLA genes are highly polymorphic, and their association with a specific disease has been used as evidence to suggest the involvement of immunological mechanisms in pathogenesis. HLA genes are divided into A, B, and C, which encode MHC class 1 molecules, and DP, DQ, and DR, which encode MHC class II molecules.

A Taiwanese study investigated genetic polymorphisms of MHC class I molecules and found that the allele frequencies of the B*39:01 and C*03:02 genes were significantly increased in clinic-based migraine compared to those in controls with odd ratios (OR) of 1.80 (95% confidence interval [CI] = 1.28–2.53) and 1.50 (95% CI = 1.14–1.62), respectively [99]. The study also found that allele frequencies of C*03:02 were significantly increased in individuals with CM with medication-overuse headache compared to those in individuals with EM, with an OR of 1.63 (95% CI = 1.11–2.39). Regarding genetic polymorphisms in MHC class II molecules, an Italian study found a decreased frequency of the DRB1*12 allele and an increased frequency of the DRB1*16 allele in patients with migraine compared to that in controls. Furthermore, a subgroup analysis revealed a significant increase in the DRB1*16 allele in MO, but not in MA [100]. Another Italian study reported an increased frequency of DRB1*15 and DRB1*16 alleles in individuals with MA compared to that in those with MO [101].

#### Cytokine gene polymorphism

Among cytokine gene polymorphisms, TNF-α 308G > A is a genetic polymorphism in migraine for which a significant relationship is frequently reported. In a meta-analysis of 11 studies including 6,682 Caucasians and 22,591 non-Caucasians, the A allele (AA + GA vs. GG) in TNF-α 308G > A was associated with an increased OR of 1.82 (95% CI = 1.15–2.87) for migraine in non-Caucasians, but no significant difference between migraine and control groups of Caucasians (OR = 0.81, 95% CI = 0.56–1.17) [102]. When comparing MA with controls, the risk was significantly increased for both Caucasians (OR = 1.15, 95% CI = 1.03–1.28) and non-Caucasians (OR = 1.62, 95% CI = 1.303–2.53). Other reported cytokine gene polymorphisms associated with migraine include TNF-β G252A, rs1800629, rs1799724, and rs1799724 polymorphisms in the TNF-α gene and the C/T biallelic polymorphism IL-1α gene [103–106].

#### Other polymorphisms related to immunity

A meta-analysis including 375,000 individuals from 22 genome-wide association studies identified 38 loci for migraine, and some loci were associated with immune mechanisms, including rs67914 near *TGFBR2*, rs7684253 near *REST-SPINK2*, rs28455731 near *GJA1*, *YAP1*, *PRDM16, LRP1*, and *MRV11* [10, 107].

In summary, genetic polymorphisms linked to immune dysfunction have been reported in patients with migraine; however, conflicting results have been reported in most cases. One genetic variation for which a relatively large number of studies report a significant association is the A allele in TNF-α 308G > A. Although the number of studies on the association between migraine and HLA gene polymorphisms is small, this is an important finding that suggests the involvement of immunological mechanisms in migraine.

### Comorbidity with autoimmune and allergic diseases

#### Migraine and autoimmune diseases

Altered cytokine profiles and lymphocyte subsets are also found in autoimmune diseases such as migraine. Studies have reported a higher prevalence of migraine in the presence of autoimmune diseases [108]. Although it remains uncertain whether migraine in autoimmune diseases is a primary headache or a symptom of autoimmune diseases, the co-occurrence of the two conditions suggests that migraine and autoimmune diseases share an immunological pathogenesis.

##### Multiple sclerosis

A meta-analysis of 11 articles and two abstracts found a high prevalence of migraine in patients with multiple sclerosis, at a rate of 31% [109]. Disease-modifying treatments affect migraine, with an increasing prevalence and worsening symptoms [110, 111].

##### Vasculitis

Behçet’s disease affects both the small and large veins, and 85.2% of patients experience headaches, with 90% headaches meeting the International Headache Society’s criteria for migraine. There is a 52% prevalence of visual or sensory auras in patients with Behçet’s disease, which is higher than that in the general population. When assessing disability with the Migraine Disability Assessment, 62% respondents had moderate or severe disability [112].

##### Connective tissue disorders

Systemic lupus erythematosus (SLE), rheumatoid arthritis, Sjögren’s disease, psoriatic arthritis, and antiphospholipid syndrome are autoimmune connective tissue disorders. Headache or migraine is more common in most autoimmune connective tissue disorders than in controls, except in SLE [113–116]. Conflicting results have been reported for headaches in patients with SLE; however, a meta-analysis of 112 studies found that headaches in patients with SLE did not differ from those in controls [117].

##### Immune-mediated gastrointestinal disorders

Celiac disease (CD) and inflammatory bowel disease (IBD) are immune-mediated gastrointestinal disorders. Individuals with CD or IBD have a higher prevalence of migraine or headaches than those without these disorders [118]. A meta-analysis of 40 articles found that the prevalence of headaches in patients with CD is 26%, with most headaches being migraine-like. A meta-analysis of 10 studies found that the pooled prevalence of migraine in patients with IBD was 19%. When analyzing ulcerative colitis alone, the prevalence of migraine was 10%, and that of Crohn’s disease was 24% [109].

#### Migraine and allergic diseases

Allergic diseases are caused by the hypersensitivity of the immune system to harmful substances. These conditions include asthma, atopic dermatitis, allergic conjunctivitis, allergic rhinitis, food allergies, and anaphylaxis.

In cross-sectional studies, individuals with migraine had a higher prevalence of hay fever, rhinitis, and dermatitis than those without migraine [119]. Similarly, the prevalence of migraine was higher in individuals with allergic rhinitis, atopic dermatitis, and asthma [120–122]. Longitudinal studies have shown a bidirectional relationship between migraine and asthma; the incidence of asthma is higher in individuals with migraine than in those without migraine, and the incidence of migraine is higher in individuals with asthma than in those without allergies [123–125]. A significant association between migraine and food allergies has also been reported [126]; however, the relationship between allergic diseases other than asthma and migraine in longitudinal studies is not well understood.

In summary, cross-sectional studies have consistently demonstrated significant comorbidity between allergic conditions, such as asthma, rhinitis, dermatitis, and food allergies, and migraine. However, in longitudinal studies, only one article reported bilateral comorbidities specifically between asthma—an allergic disease—and migraine. Despite this, other studies individually revealed an increased incidence of migraines among patients with allergies, and vice versa. These findings suggest a pattern of bidirectional comorbidity between migraines and allergic diseases.

### Other relevant findings

#### CGRP

CGRP is composed of 37 amino acids and is abundant in both the central and peripheral nervous systems. CGRP is released by transient receptor potential vanilloid 1 activation and plays an important role in migraine and immunity [9]. CGRP also plays a key role in the pathogenesis of migraine. Infusions of CGRP induce migraine-like headaches in humans and animals [127]. Ictal elevation of blood CGRP levels in migraine has been consistently reported [128]. Meanwhile, interictal CGRP levels increase in patients with CM [129]. The recent introduction of CGRP-targeted therapies has revolutionized migraine treatment and has become increasingly widely used [130].

CGRP is abundant in the immune system and exhibits both proinflammatory and anti-inflammatory effects [9, 131, 132]. Nerve fibers containing CGRP are found in various body parts, including the bone marrow, gut, lungs, lymph nodes, skin, spleen, and thymus. Moreover, CGRP receptors are present in many hematopoietic cell types [133]. Studies directly investigating the immunomodulatory effect of CGRP in patients with migraine are still scarce. The effects of CGRP on immune function, mostly from animal models, are summarized in Table 2.

**Table 2:** Effects of CGRP on immune functions

Immune systems	Effects of CGRP
Macrophages	Induce degradation of MHC class II molecules [134] Inhibit LPS-induced proinflammatory cytokines (IL-1b and TNF-α) and promote anti-inflammatory cytokines (IL-4 and IL-10) from macrophages [135, 136] Inhibit CD11b expression [137] Inhibit toll-like receptor-mediated inflammation
B cells	Inhibit early B cell development and differentiation [138, 139] Inhibit surface expression of immunoglobulin [138]
T cells	Inhibit T cell proliferation and differentiation [140, 141] Inhibit T cell motility and migration [141] Facilitate breaking of T helper cells [142]
Dendritic cells	Inhibit antigen presentation and maturation of dendritic cells [132, 143]
Mast cells	Inhibit mast cell degranulation [132, 143, 144] Most meningeal mast cells are found near CGRP-containing nerve fibers
Cytokines	Increase production of Th2 cytokines and decrease production of Th1 cytokines [9]

CGRP, calcitonin gene-related peptide; MHC, major histocompatibility complex; LPS, lipopolysaccharide

#### Pituitary adenylate cyclase-activating polypeptide (PACAP)

PACAP, like CGRP, may play an important role in the pathogenesis of migraine. PACAP exists in two isoforms, PACAP-38 and PACAP-27, with PACAP-38 being the most prevalent. Most of the immunological functional studies of PACAP have been conducted with PACAP-38.

Increased PACAP-38 levels have been observed during migraine attacks [145, 146]. During the interictal period, PACAP-38 levels showed mixed results; some studies showed increased levels, while others revealed decreased levels [145–148]. PACAP-38 infusion induces migraine-like headaches in humans [149].

PACAP-38 also affects immune function and exhibits potent anti-inflammatory effects, inhibiting the production of proinflammatory cytokines such as IL-2, IL-12, and TNF-α in macrophages and stimulating the production of anti-inflammatory cytokines such as IL-10 [150–153]. PACAP-38 inhibits antigen presentation by macrophages by inhibiting the B7.1 accessory molecule and increasing Th2 differentiation and decreasing Th1 differentiation [154].

## Discussion

**Fig. 2:**
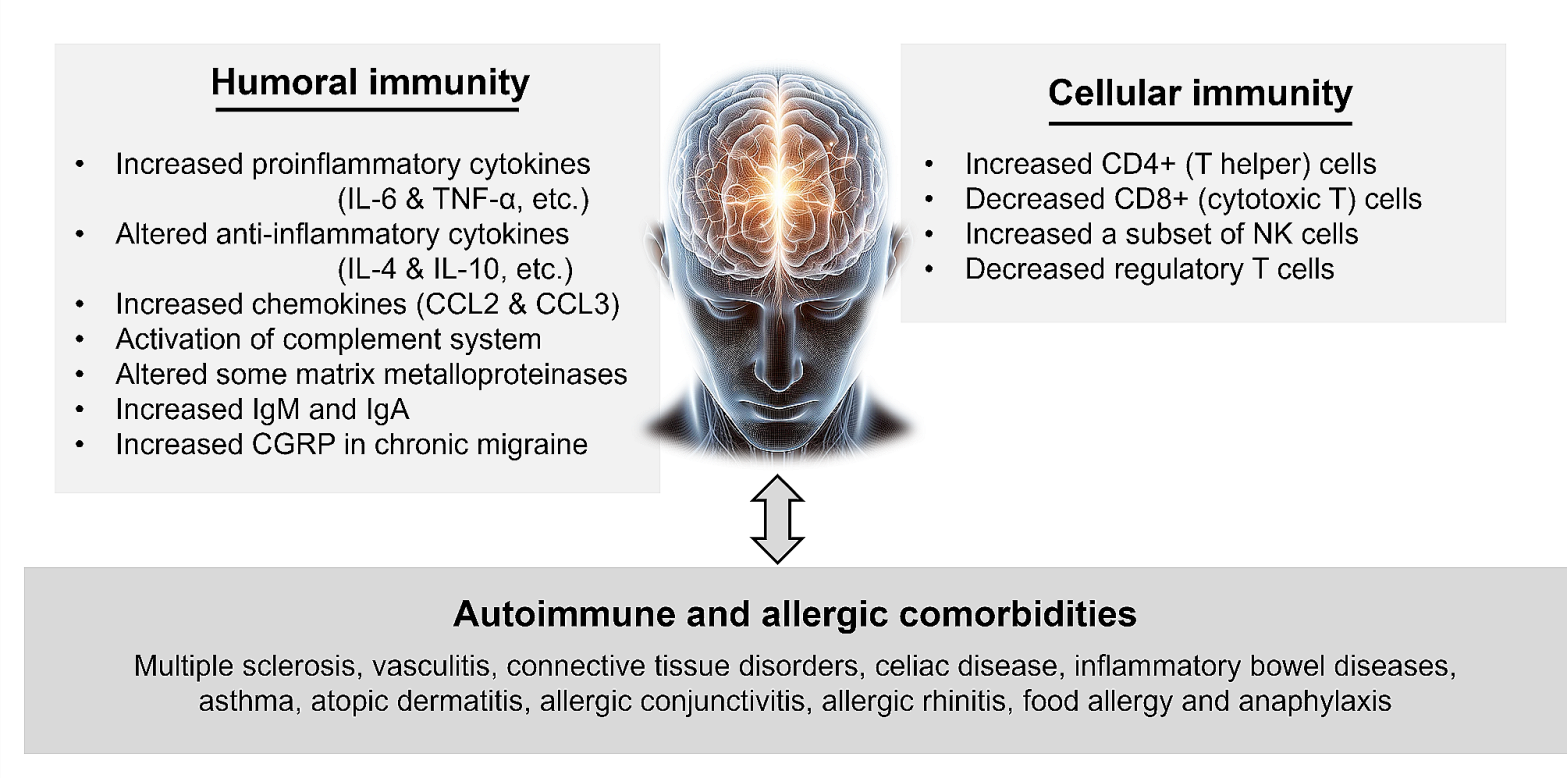
The immunological changes in migraine during the interictal period. CCL, chemokine ligand; NK, natural killer; CGRP, calcitonin gene-related peptide; PACAP, pituitary adenylate cyclase-activating polypeptide

Although the immunological changes in migraine vary across studies and the timing of the attack, the key findings that are relatively consistent can be summarized as follows: (1) some proinflammatory cytokines, such as IL-1b, IL-6 and TNF-α, increase during the interictal or ictal period, while anti-inflammatory cytokines show various findings; (2) an increase in T helper lymphocytes, a decrease in cytotoxic T lymphocytes, and a decrease in regulatory T lymphocytes are observed; and (3) there is significant comorbidity with autoimmune and allergic diseases where immunological mechanisms play an important role in the pathogenesis. The immunological changes in migraine during the interictal period are summarized in Fig. 2.

Allergic diseases, in which immunological mechanisms play an important role in disease pathogenesis, are co-morbid with migraine [119–126]. This comorbidity suggests that dysfunction of the immunological system is a common pathological mechanism in both migraine and allergic diseases. As each allergic disease is caused by different immunological mechanisms, evaluating the comorbidity of allergic diseases and migraine separately according to the types of the allergic reaction will provide a better understanding of the immunological aspects of migraine. The comorbidity between autoimmune diseases and migraine has not been as thoroughly investigated as that with allergic diseases [108, 109, 112, 117, 118]. While many studies have noted a higher prevalence of migraine among individuals with autoimmune diseases, there is still scant information regarding the prevalence of autoimmune conditions in those suffering from migraine.

We observed a variety of immune response changes in humans suffering from migraine, but some of the changes observed in preclinical studies were different from those observed in animal models. An activation of meningeal macrophages, DCs, and MCs has been documented in animal models of migraine [1]. However, macrophage activation has not been observed in human migraine studies [155]. CGRP receptors have been observed in rat MCs, but no CGRP receptors have been identified in human MCs [156, 157]. This is thought to be due to the differences between animal models and human migraine, as well as species differences. Animal models can be used to observe pathological changes in various tissues and organs that cannot be observed in humans. Therefore, it is important to note that findings using animal models of migraine may differ from those of human migraine; therefore, caution should be exercised when interpreting studies using animal models of migraine.

In this review, we explored the immunological role of CGRP, present in both the nervous and non-nervous systems of the body, performing a multitude of physiological functions. Furthermore, it plays a significant role in the pathophysiology of various diseases, including ischemia, heart failure, atherosclerosis, arthritis, sepsis, hypertension, diabetes, and obesity [158]. In terms of its immunological function, CGRP primarily acts to suppress inflammation, by inhibiting proinflammatory Th1 cytokines, increasing anti-inflammatory Th2 cytokines, and suppressing immune cells such as macrophages, B cells, T cells, DCs, and mast cells [9, 132, 134–141, 143, 144]. Given that immunological changes associated with migraine typically involve increased inflammation—characterized by heightened levels of proinflammatory cytokines, an increase in T helper cells, and a reduction in regulatory T cells—CGRP appears to play a pivotal role in immunoregulation within the context of migraine. Similarly, PACAP is believed to be involved in migraine pathogenesis and possesses immunomodulatory properties. These properties of PACAP also contribute to the suppression of inflammation, achieved through the inhibition of proinflammatory cytokines and the enhancement of anti-inflammatory cytokines [145–154]. However, the precise roles of CGRP and PACAP in immune alterations associated with migraine remain insufficiently explored, highlighting the need for future research.

We reviewed the various immunological alterations observed in patients with migraine. Recently, various therapeutic approaches have been developed to modulate these immunological alterations. Corticosteroids, immunoglobulins, and cytotoxic agents have traditionally been used to treat altered immunity in autoimmune diseases. More recently, monoclonal antibodies directed against specific immune function cells or substances, calcineurin inhibitors, tacrolimus for selective suppression of T lymphocytes, and sirolimus and everolimus have been used to inhibit the mammalian target of rapamycin [159]. If immunological mechanisms play a key role in migraine and their targets can be identified, it may be possible to develop effective migraine therapies using immunomodulation. Therefore, identifying immune alterations in migraine may help guide future targeted immunomodulatory therapies.

This study has a few limitations. First, our systematic search of the PubMed database aimed to identify immunological changes in migraine. However, capturing the full diversity of immune responses in human migraines is challenging. As a result, many studies addressing immunological changes in migraine were included through additional searches beyond our initial strategy, which we believe was necessary given the diverse nature of immune responses. Second, various factors can affect immune function. These include the timing and location of blood sample collection, the speed of the analysis, the presence of comorbidities, nutritional status, and medication use. These heterogeneities may have contributed to the inconsistent results of the studies. Furthermore, the limited number of studies that could use the quantitative values of the measurements also restricted the quality of meta-analysis. Due to this, we aimed to focus on both quantitative and qualitative comparisons of the immunological changes.

## Conclusions

Altered immunity is noted during the ictal and interictal periods in patients with migraine. A pivotal mechanism of migraine is the activation and sensitization of trigeminal vascular afferents and the subsequent neuroinflammatory responses. Various immunological changes in migraine are thought to be closely associated with neuroinflammatory responses. The main immunological changes in human migraine include increased blood levels of some proinflammatory cytokines, adipokines, and CGRP; activation of the complement system; an increase in Th lymphocytes; and a decrease in Treg lymphocytes. The comorbidity with autoimmune and allergic diseases also suggests other immunological mechanisms in migraine. These identifications not only contribute to our understanding of pathophysiology but may also aid in the diagnosis and treatment of migraine, reducing the burden thereof.

### Electronic supplementary material

Below is the link to the electronic supplementary material.

Supplementary Material 1

## Data Availability

The data extracted for the analyses are available upon reasonable request from the corresponding author.

## Abbreviations

ADP: Adiponectin
CAMs: Cell adhesion molecules
CD: Celiac disease
CGRP: Calcitonin gene-related peptide
CM: Chronic migraine
COX-2: Cyclooxygenase-2
CSF: Cerebrospinal fluid
DC: Dendritic cells
EM: Episodic migraine
HLA: Human lymphocyte antigen
hsCRP: High sensitivity C-reactive protein
IBD: inflammatory bowel disease
ICAM-1: Intercellular adhesion molecule-1
INF-γ: Interferon-γ
IL: Interleukin
MA: Migraine with aura
MC: Mast cells
MHC: Major histocompatibility complex
MMPs: Matrix metalloproteinases
MO: Migraine without aura
NK: Natural killer
PACAP: Pituitary adenylate cyclase-activating polypeptide
PTX-3: Pentraxin-3
SLE: Systemic lupus erythematosus
TGF-β: Transforming growth factor-β
TNF-α: Tumor necrosis factor-α
TRPV-1: Transient receptor potential vanilloid-1

## References

[1] Biscetti L, Cresta E, Cupini LM, Calabresi P, Sarchielli P (2023). The putative role of neuroinflammation in the complex pathophysiology of migraine: from bench to bedside. Neurobiol Dis.

[2] Malhotra R (2016). Understanding migraine: potential role of neurogenic inflammation. Ann Indian Acad Neurol.

[3] Balcziak LK, Russo AF (2022). Dural Immune Cells, CGRP, and Migraine. Front Neurol.

[4] Pradalier A, Launay JM (1996). Immunological aspects of migraine. Biomed Pharmacother.

[5] Moskowitz MA (1993). Neurogenic inflammation in the pathophysiology and treatment of migraine. Neurology.

[6] Cairns BE, Arendt-Nielsen L, Sacerdote P (2015). Perspectives in Pain Research 2014: Neuroinflammation and glial cell activation: the cause of transition from acute to chronic pain?. Scand J Pain.

[7] Levy D, Labastida-Ramirez A, MaassenVanDenBrink A (2019). Current understanding of meningeal and cerebral vascular function underlying migraine headache. Cephalalgia.

[8] Hosoi J, Murphy GF, Egan CL, Lerner EA, Grabbe S, Asahina A, Granstein RD (1993). Regulation of Langerhans cell function by nerves containing calcitonin gene-related peptide. Nature.

[9] Assas BM, Pennock JI, Miyan JA (2014). Calcitonin gene-related peptide is a key neurotransmitter in the neuro-immune axis. Front Neurosci.

[10] Biscetti L, De Vanna G, Cresta E, Bellotti A, Corbelli I, Letizia Cupini M, Calabresi P, Sarchielli P (2022). Immunological findings in patients with migraine and other primary headaches: a narrative review. Clin Exp Immunol.

[11] Aczél T, Benczik B, Ágg B, Körtési T, Urbán P, Bauer W, Gyenesei A, Tuka B, Tajti J, Ferdinandy P, Vécsei L, Bölcskei K, Kun J, Helyes Z (2022). Disease- and headache-specific microRNA signatures and their predicted mRNA targets in peripheral blood mononuclear cells in migraineurs: role of inflammatory signalling and oxidative stress. J Headache Pain.

[12] Tricco AC, Lillie E, Zarin W, O’Brien KK, Colquhoun H, Levac D, Moher D, Peters MDJ, Horsley T, Weeks L, Hempel S, Akl EA, Chang C, McGowan J, Stewart L, Hartling L, Aldcroft A, Wilson MG, Garritty C, Lewin S, Godfrey CM, Macdonald MT, Langlois EV, Soares-Weiser K, Moriarty J, Clifford T, Tunçalp Ö, Straus SE (2018). PRISMA Extension for scoping reviews (PRISMA-ScR): Checklist and Explanation. Ann Intern Med.

[13] Haddaway NR, Page MJ, Pritchard CC, McGuinness LA (2022). PRISMA2020: an R package and Shiny app for producing PRISMA 2020-compliant flow diagrams, with interactivity for optimised digital transparency and open synthesis. Campbell Syst Reviews.

[14] Wells GAS, O’Connell B, Peterson D, Welch J, Losos V, Tugwell M (2000) P. The Newcastle-Ottawa Scale (NOS) for assessing the quality of nonrandomised studies in meta-analyses. https://www.ohri.ca/programs/clinical_epidemiology/oxford.asp. Accessed 2024 Feb 2

[15] Higgins J, Li T, Deeks J (2023) Chap. 6: Choosing effect measures and computing estimates of effect. In: Higgins J, Thomas J, Chandler J, Cumpston M, Li T, Page M, Welch V (eds) Cochrane Handbook for Systematic Reviews of Interventions version 6.4 (updated August 2023). Cochrane

[16] Chaudhry SR, Lendvai IS, Muhammad S, Westhofen P, Kruppenbacher J, Scheef L, Boecker H, Scheele D, Hurlemann R, Kinfe TM (2019). Inter-ictal assay of peripheral circulating inflammatory mediators in migraine patients under adjunctive cervical non-invasive vagus nerve stimulation (nVNS): a proof-of-concept study. Brain Stimul.

[17] Hirfanoglu T, Serdaroglu A, Gulbahar O, Cansu A (2009). Prophylactic drugs and cytokine and leptin levels in children with migraine. Pediatr Neurol.

[18] ArmaĞAn HH, Karaman K, YalÇIn Yilmaz D (2020). Antioxidant and cytokine levels in plasma of patients with attack and non-attack periods. J Cell Neurosci Oxidative Stress.

[19] Lee YY, Yang YP, Huang PI, Li WC, Huang MC, Kao CL, Chen YJ, Chen MT (2015). Exercise suppresses COX-2 pro-inflammatory pathway in vestibular migraine. Brain Res Bull.

[20] Uzar E, Evliyaoglu O, Yucel Y, Ugur Cevik M, Acar A, Guzel I, Islamoglu Y, Colpan L, Tasdemir N (2011). Serum cytokine and pro-brain natriuretic peptide (BNP) levels in patients with migraine. Eur Rev Med Pharmacol Sci.

[21] Yücel M, Kotan D, Gurol Çiftçi G, Çiftçi IH, Cikriklar HI (2016). Serum levels of endocan, claudin-5 and cytokines in migraine. Eur Rev Med Pharmacol Sci.

[22] Flook M, Frejo L, Gallego-Martinez A, Martin-Sanz E, Rossi-Izquierdo M, Amor-Dorado JC, Soto-Varela A, Santos-Perez S, Batuecas-Caletrio A, Espinosa-Sanchez JM, Pérez-Carpena P, Martinez-Martinez M, Aran I, Lopez-Escamez JA (2019). Differential Proinflammatory signature in vestibular migraine and Meniere Disease. Front Immunol.

[23] Karaaslan Z, Özçelik P, Ulukan Ç, Ulusoy C, Orhan KS, Orhan EK, Küçükali C, Tüzün E, Baykan B, Akdal G (2020). Plasma levels of inflammatory mediators in vestibular migraine. Int J Neurosci.

[24] Mueller L, Gupta AK, Stein TP (2001). Deficiency of tumor necrosis factor alpha in a subclass of menstrual migraineurs. Headache.

[25] Oliveira AB, Bachi ALL, Ribeiro RT, Mello MT, Tufik S, Peres MFP (2017). Unbalanced plasma TNF-α and IL-12/IL-10 profile in women with migraine is associated with psychological and physiological outcomes. J Neuroimmunol.

[26] Perini F, D’Andrea G, Galloni E, Pignatelli F, Billo G, Alba S, Bussone G, Toso V (2005). Plasma cytokine levels in migraineurs and controls. Headache.

[27] Fidan I, Yüksel S, Ymir T, Irkeç C, Aksakal FN (2006). The importance of cytokines, chemokines and nitric oxide in pathophysiology of migraine. J Neuroimmunol.

[28] Sarchielli P, Alberti A, Baldi A, Coppola F, Rossi C, Pierguidi L, Floridi A, Calabresi P (2006). Proinflammatory cytokines, adhesion molecules, and lymphocyte integrin expression in the internal jugular blood of migraine patients without aura assessed ictally. Headache.

[29] van Hilten JJ, Ferrari MD, Van der Meer JW, Gijsman HJ, Looij BJ (1991). Plasma interleukin-1, tumour necrosis factor and hypothalamic-pituitary-adrenal axis responses during migraine attacks. Cephalalgia.

[30] Aydın M, Demir C, Arıkanoğlu A, Bulut S, Ilhan N (2015). Plasma cytokine levels in migraineurs during and outside of attacks. Electron J Gen Med.

[31] Domínguez C, Vieites-Prado A, Pérez-Mato M, Sobrino T, Rodríguez-Osorio X, López A, Campos F, Martínez F, Castillo J, Leira R (2018). CGRP and PTX3 as predictors of efficacy of Onabotulinumtoxin Type A in Chronic Migraine: an observational study. Headache.

[32] Koçer A, Memişoğullari R, Domaç FM, Ilhan A, Koçer E, Okuyucu S, Ozdemir B, Yüksel H (2009). IL-6 levels in migraine patients receiving topiramate. Pain Pract.

[33] Togha M, Razeghi Jahromi S, Ghorbani Z, Ghaemi A, Rafiee P (2020). Evaluation of inflammatory state in migraineurs: a case-control study. Iran J Allergy Asthma Immunol.

[34] Wang F, He Q, Ren Z, Li F, Chen W, Lin X, Zhang H, Tai G (2015). Association of serum levels of intercellular adhesion molecule-1 and interleukin-6 with migraine. Neurol Sci.

[35] Cowan RP, Gross NB, Sweeney MD, Sagare AP, Montagne A, Arakaki X, Fonteh AN, Zlokovic BV, Pogoda JM, Harrington MG (2021). Evidence that blood-CSF barrier transport, but not inflammatory biomarkers, change in migraine, while CSF sVCAM1 associates with migraine frequency and CSF fibrinogen. Headache.

[36] Empl M, Sostak P, Riedel M, Schwarz M, Müller N, Förderreuther S, Straube A (2003). Decreased sTNF-RI in migraine patients?. Cephalalgia.

[37] Martelletti P, Stirparo G, Rinaldi C, Frati L, Giacovazzo M (1993). Disruption of the immunopeptidergic network in dietary migraine. Headache.

[38] Martami F, Razeghi Jahromi S, Togha M, Ghorbani Z, Seifishahpar M, Saidpour A (2018). The serum level of inflammatory markers in chronic and episodic migraine: a case-control study. Neurol Sci.

[39] Boćkowski L, Sobaniec W, Zelazowska-Rutkowska B (2009). Proinflammatory plasma cytokines in children with migraine. Pediatr Neurol.

[40] Guo S, Vollesen AL, Hansen YB, Frandsen E, Andersen MR, Amin FM, Fahrenkrug J, Olesen J, Ashina M (2017). Part II: biochemical changes after pituitary adenylate cyclase-activating polypeptide-38 infusion in migraine patients. Cephalalgia.

[41] Tanure MT, Gomez RS, Hurtado RC, Teixeira AL, Domingues RB (2010). Increased serum levels of brain-derived neurotropic factor during migraine attacks: a pilot study. J Headache Pain.

[42] Munno I, Centonze V, Marinaro M, Bassi A, Lacedra G, Causarano V, Nardelli P, Cassiano MA, Albano O (1998). Cytokines and migraine: increase of IL-5 and IL-4 plasma levels. Headache.

[43] Martelletti P, Stirparo G, Morrone S, Rinaldi C, Giacovazzo M (1997). Inhibition of intercellular adhesion molecule-1 (ICAM-1), soluble ICAM-1 and interleukin-4 by nitric oxide expression in migraine patients. J Mol Med (Berl).

[44] Ishizaki K, Takeshima T, Fukuhara Y, Araki H, Nakaso K, Kusumi M, Nakashima K (2005). Increased plasma transforming growth factor-beta1 in migraine. Headache.

[45] Güzel I, Taşdemir N, Celik Y (2013). Evaluation of serum transforming growth factor β1 and C-reactive protein levels in migraine patients. Neurol Neurochir Pol.

[46] Shimomura T, Araga S, Esumi E, Takahashi K (1991). Decreased serum interleukin-2 level in patients with chronic headache. Headache.

[47] Duarte H, Teixeira AL, Rocha NP, Domingues RB (2015). Increased interictal serum levels of CXCL8/IL-8 and CCL3/MIP-1α in migraine. Neurol Sci.

[48] Sarchielli P, Alberti A, Vaianella L, Pierguidi L, Floridi A, Mazzotta G, Floridi A, Gallai V (2004). Chemokine levels in the jugular venous blood of migraine without aura patients during attacks. Headache.

[49] Domingues RB, Duarte H, Senne C, Bruniera G, Brunale F, Rocha NP, Teixeira AL (2016). Serum levels of adiponectin, CCL3/MIP-1α, and CCL5/RANTES discriminate migraine from tension-type headache patients. Arq Neuropsiquiatr.

[50] Taylor EB (2021). The complex role of adipokines in obesity, inflammation, and autoimmunity. Clin Sci (Lond).

[51] Peterlin BL, Alexander G, Tabby D, Reichenberger E (2008). Oligomerization state-dependent elevations of adiponectin in chronic daily headache. Neurology.

[52] Peterlin BL, Sacco S, Bernecker C, Scher AI (2016). Adipokines and migraine: a systematic review. Headache.

[53] Chai NC, Gelaye B, Tietjen GE, Dash PD, Gower BA, White LW, Ward TN, Scher AI, Peterlin BL (2015). Ictal adipokines are associated with pain severity and treatment response in episodic migraine. Neurology.

[54] Bernecker C, Pailer S, Kieslinger P, Horejsi R, Möller R, Lechner A, Wallner-Blazek M, Weiss S, Fazekas F, Truschnig-Wilders M, Gruber HJ (2011). Increased matrix metalloproteinase activity is associated with migraine and migraine-related metabolic dysfunctions. Eur J Neurol.

[55] Bernecker C, Ragginer C, Fauler G, Horejsi R, Möller R, Zelzer S, Lechner A, Wallner-Blazek M, Weiss S, Fazekas F, Bahadori B, Truschnig-Wilders M, Gruber HJ (2011). Oxidative stress is associated with migraine and migraine-related metabolic risk in females. Eur J Neurol.

[56] Dearborn JL, Schneider AL, Gottesman RF, Kurth T, Pankow JS, Couper DJ, Rose KM, Williams MA, Peterlin BL (2014). Adiponectin and leptin levels in migraineurs in the atherosclerosis risk in communities Study. Neurology.

[57] Duarte H, Teixeira AL, Rocha NP, Domingues RB (2014). Increased serum levels of adiponectin in migraine. J Neurol Sci.

[58] Guldiken B, Guldiken S, Demir M, Turgut N, Tugrul A (2008). Low leptin levels in migraine: a case control study. Headache.

[59] Ligong Z, Jinjin Q, Chunfu C, Congcong L, Xiaojun D (2015). Effect of obesity and leptin level on migraineurs. Med Sci Monit.

[60] Elkington PT, O’Kane CM, Friedland JS (2005). The paradox of matrix metalloproteinases in infectious disease. Clin Exp Immunol.

[61] Ashina M, Tvedskov JF, Lipka K, Bilello J, Penkowa M, Olesen J (2010). Matrix metalloproteinases during and outside of migraine attacks without aura. Cephalalgia.

[62] Imamura K, Takeshima T, Fusayasu E, Nakashima K (2008). Increased plasma matrix metalloproteinase-9 levels in migraineurs. Headache.

[63] Park CG, Lee SH, Chu MK (2022). No change in interictal C-reactive protein levels in individuals with episodic and chronic migraine: a case-control study and literature review. Front Neurol.

[64] Gudmundsson LS, Aspelund T, Scher AI, Thorgeirsson G, Johannsson M, Launer LJ, Gudnason V (2009). C-reactive protein in migraine sufferers similar to that of non-migraineurs: the Reykjavik Study. Cephalalgia.

[65] Welch KM, Brandes AW, Salerno L, Brandes JL (2006). C-reactive protein may be increased in migraine patients who present with complex clinical features. Headache.

[66] Lord GD, Duckworth JW (1978). Complement and immune complex studies in migraine. Headache.

[67] Lord GD, Duckworth JW, Charlesworth JA (1977). Complement activation in migraine. Lancet.

[68] Lord GD, Duckworth JW (1977). Immunoglobulin and complement studies in migraine. Headache.

[69] Behan WMH, Behan PO, Durward WF (1981) Complement Studies in Migraine. Headache: The Journal of Head and Face Pain 21(2):55–57. 10.1111/j.1526-4610.1981.hed2102055.x 10.1111/j.1526-4610.1981.hed2102055.x 7239901

[70] Jerzmanowski A, Klimek A (1983). Immunoglobulins and complement in migraine. Cephalalgia.

[71] Shimomura T, Araga S, Kowa H, Takahashi K (1992). Immunoglobulin kappa/lambda ratios in migraine and tension-type headache. Jpn J Psychiatry Neurol.

[72] Gazerani P, Pourpak Z, Ahmadiani A, Hemmati A, Kazemnejad A (2003). A correlation between migraine, histamine and immunoglobulin e. Scand J Immunol.

[73] Park CG, Na HY, Park DE, Kim HY, Chu MK (2023). Altered interictal serum histamine and immunoglobulin E but unchanged tryptase levels in individuals with episodic and chronic migraine. Cephalalgia.

[74] Moghadam MH, Ardalani H, Alehashemi A, Dehkordi MA, Meshkat M (2016). Correlation between severity of migraine attacks and IgE level in peripheral blood. LaboratoriumsMedizin.

[75] Worm J, Falkenberg K, Olesen J (2019). Histamine and migraine revisited: mechanisms and possible drug targets. J Headache Pain.

[76] Koyuncu Irmak D, Kilinc E, Tore F (2019). Shared Fate of Meningeal mast cells and sensory neurons in Migraine. Front Cell Neurosci.

[77] Cacabelos R, Torrellas C, Fernández-Novoa L, Aliev G (2016). Neuroimmune Crosstalk in CNS disorders: the histamine connection. Curr Pharm Des.

[78] Haimart M, Pradalier A, Launay JM, Dreux C, Dry J (1987). Whole blood and plasma histamine in common migraine. Cephalalgia.

[79] Heatley RV, Denburg JA, Bayer N, Bienenstock J (1982). Increased plasma histamine levels in migraine patients. Clin Allergy.

[80] Vural S, Albayrak L (2022). Can calcitonin gene-related peptide (CGRP) and pentraxin-3 (PTX-3) be useful in diagnosing acute migraine attack?. J Recept Signal Transduct Res.

[81] Gokdemir MT, Nas C, Gokdemir GS (2020). Pentraxin 3 level in acute migraine attack with aura: patient management in the emergency department. Am J Emerg Med.

[82] Ceylan M, Bayraktutan OF, Becel S, Atis Ö, Yalcin A, Kotan D (2016). Serum levels of pentraxin-3 and other inflammatory biomarkers in migraine: Association with migraine characteristics. Cephalalgia.

[83] Domínguez-Vivero C, Leira Y, López-Ferreiro A, Saavedra M, Rodríguez-Osorio X, Sobrino T, Campos F, Castillo J, Leira R (2020) Pentraxin 3 (PTX3): a molecular marker of endothelial dysfunction in chronic migraine. J Clin Med 9(3). 10.3390/jcm9030849 10.3390/jcm9030849 PMC7141491 32244987

[84] Li C, Zhu Q, He Q, Wang J, Wang F, Zhang H (2017). Plasma levels of Cyclooxygenase-2 (COX-2) and Visfatin during different stages and different subtypes of Migraine headaches. Med Sci Monit.

[85] Brown PD, Davies SL, Speake T, Millar ID (2004). Molecular mechanisms of cerebrospinal fluid production. Neuroscience.

[86] Waage A, Halstensen A, Shalaby R, Brandtzaeg P, Kierulf P, Espevik T (1989). Local production of tumor necrosis factor alpha, interleukin 1, and interleukin 6 in meningococcal meningitis. Relation to the inflammatory response. J Exp Med.

[87] Bø SH, Davidsen EM, Gulbrandsen P, Dietrichs E, Bovim G, Stovner LJ, White LR (2009). Cerebrospinal fluid cytokine levels in migraine, tension-type headache and cervicogenic headache. Cephalalgia.

[88] Rozen T, Swidan SZ (2007). Elevation of CSF tumor necrosis factor alpha levels in new daily persistent headache and treatment refractory chronic migraine. Headache.

[89] Sarchielli P, Alberti A, Floridi A, Gallai V (2001). Levels of nerve growth factor in cerebrospinal fluid of chronic daily headache patients. Neurology.

[90] Gallai V, Alberti A, Gallai B, Coppola F, Floridi A, Sarchielli P (2003). Glutamate and nitric oxide pathway in chronic daily headache: evidence from cerebrospinal fluid. Cephalalgia.

[91] Sarchielli P, Pini LA, Coppola F, Rossi C, Baldi A, Mancini ML, Calabresi P (2007). Endocannabinoids in chronic migraine: CSF findings suggest a system failure. Neuropsychopharmacology.

[92] van Dongen RM, Zielman R, Noga M, Dekkers OM, Hankemeier T, van den Maagdenberg AM, Terwindt GM, Ferrari MD (2017). Migraine biomarkers in cerebrospinal fluid: a systematic review and meta-analysis. Cephalalgia.

[93] Kemper RH, Meijler WJ, Korf J, Ter Horst GJ (2001). Migraine and function of the immune system: a meta-analysis of clinical literature published between 1966 and 1999. Cephalalgia.

[94] Empl M, Sostak P, Breckner M, Riedel M, Müller N, Gruber R, Förderreuther S, Straube A (1999). T-cell subsets and expression of integrins in peripheral blood of patients with migraine. Cephalalgia.

[95] Leone M, Biffi M, Leoni F, Bussone G (1994). Leukocyte subsets and cortisol serum levels in patients with migraine without aura and chronic tension-type headache. Cephalalgia.

[96] Li H, Fu Q, Philips K, Sun Y, Faurot KR, Gaylord SA, Mann JD (2022). Leukocyte inflammatory phenotype and function in migraine patients compared with matched non-migraine volunteers: a pilot study. BMC Neurol.

[97] Mosnaim AD, Kulaga H, Adams AJ, Wolf ME, Puente J, Freitag F, Diamond S (1998). Flow cytometric analysis of lymphocyte subsets in migraine patients during and outside of an acute headache attack. Cephalalgia.

[98] Faraji F, Shojapour M, Farahani I, Ganji A, Mosayebi G (2021). Reduced regulatory T lymphocytes in migraine patients. Neurol Res.

[99] Huang C, Chen SP, Huang YH, Chen HY, Wang YF, Lee MH, Wang SJ (2020). HLA class I alleles are associated with clinic-based migraine and increased risks of chronic migraine and medication overuse. Cephalalgia.

[100] Rainero I, Fasano E, Rubino E, Rivoiro C, Valfrè W, Gallone S, Savi L, Gentile S, Lo Giudice R, De Martino P, Dall’Omo AM, Pinessi L (2005). Association between migraine and HLA-DRB1 gene polymorphisms. J Headache Pain.

[101] Olerup O, Aldener A, Fogdell A (1993). HLA-DQB1 and -DQA1 typing by PCR amplification with sequence-specific primers (PCR-SSP) in 2 hours. Tissue Antigens.

[102] Gu L, Yan Y, Long J, Su L, Hu Y, Chen Q, Xie J, Wu G (2012). The TNF-α-308G/A polymorphism is associated with migraine risk: a meta-analysis. Exp Ther Med.

[103] Ishii M, Usami S, Hara H, Imagawa A, Masuda Y, Shimizu S (2014). MAOA and TNF-β gene polymorphisms are associated with photophobia but not osmophobia in patients with migraine. Acta Neurol Taiwan.

[104] Oikari LE, Stuart S, Okolicsanyi RK, Cox HC, Dixit S, Lea RA, Haupt LM, Griffiths LR (2013). Investigation of lymphotoxin α genetic variants in migraine. Gene.

[105] Hamad N, Alzoubi KH, Swedan SF, Khabour OF, El-Salem K (2021). Association between tumor necrosis factor alpha and lymphotoxin alpha gene polymorphisms and migraine occurrence among jordanians. Neurol Sci.

[106] Rainero I, Pinessi L, Salani G, Valfrè W, Rivoiro C, Savi L, Gentile S, Giudice RL, Grimaldi LM (2002). A polymorphism in the interleukin-1alpha gene influences the clinical features of migraine. Headache.

[107] Gormley P, Anttila V, Winsvold BS, Palta P, Esko T, Pers TH, Farh KH, Cuenca-Leon E, Muona M, Furlotte NA, Kurth T, Ingason A, McMahon G, Ligthart L, Terwindt GM, Kallela M, Freilinger TM, Ran C, Gordon SG, Stam AH, Steinberg S, Borck G, Koiranen M, Quaye L, Adams HH, Lehtimäki T, Sarin AP, Wedenoja J, Hinds DA, Buring JE, Schürks M, Ridker PM, Hrafnsdottir MG, Stefansson H, Ring SM, Hottenga JJ, Penninx BW, Färkkilä M, Artto V, Kaunisto M, Vepsäläinen S, Malik R, Heath AC, Madden PA, Martin NG, Montgomery GW, Kurki MI, Kals M, Mägi R, Pärn K, Hämäläinen E, Huang H, Byrnes AE, Franke L, Huang J, Stergiakouli E, Lee PH, Sandor C, Webber C, Cader Z, Muller-Myhsok B, Schreiber S, Meitinger T, Eriksson JG, Salomaa V, Heikkilä K, Loehrer E, Uitterlinden AG, Hofman A, van Duijn CM, Cherkas L, Pedersen LM, Stubhaug A, Nielsen CS, Männikkö M, Mihailov E, Milani L, Göbel H, Esserlind AL, Christensen AF, Hansen TF, Werge T, Kaprio J, Aromaa AJ, Raitakari O, Ikram MA, Spector T, Järvelin MR, Metspalu A, Kubisch C, Strachan DP, Ferrari MD, Belin AC, Dichgans M, Wessman M, van den Maagdenberg AM, Zwart JA, Boomsma DI, Smith GD, Stefansson K, Eriksson N, Daly MJ, Neale BM, Olesen J, Chasman DI, Nyholt DR, Palotie A (2016). Meta-analysis of 375,000 individuals identifies 38 susceptibility loci for migraine. Nat Genet.

[108] Cavestro C, Ferrero M (2018). Migraine in systemic Autoimmune diseases. Endocr Metab Immune Disord Drug Targets.

[109] Olfati H, Mirmosayyeb O, Hosseinabadi AM, Ghajarzadeh M (2023). The prevalence of Migraine in Inflammatory Bowel Disease, a systematic review and Meta-analysis. Int J Prev Med.

[110] Patti F, Nicoletti A, Pappalardo A, Castiglione A, Lo Fermo S, Messina S, D’Amico E, Cimino V, Zappia M (2012). Frequency and severity of headache is worsened by Interferon-β therapy in patients with multiple sclerosis. Acta Neurol Scand.

[111] Gebhardt M, Kropp P, Hoffmann F, Zettl UK (2019). Headache in the course of multiple sclerosis: a prospective study. J Neural Transm (Vienna).

[112] Kidd D (2006). The prevalence of headache in Behçet’s syndrome. Rheumatology (Oxford).

[113] Peri Y, Agmon-Levin N, Theodor E, Shoenfeld Y (2012). Sjögren’s syndrome, the old and the new. Best Pract Res Clin Rheumatol.

[114] Goldberg NC, Duncan SC, Winkelmann RK (1978). Migraine and systemic scleroderma. Arch Dermatol.

[115] Uluduz D, Tavsanli ME, Uygunoğlu U, Saip S, Kasapcopur O, Ozge A, Temel GO (2014). Primary headaches in pediatric patients with chronic rheumatic disease. Brain Dev.

[116] Schofield JR, Blitshteyn S, Shoenfeld Y, Hughes GR (2014). Postural tachycardia syndrome (POTS) and other autonomic disorders in antiphospholipid (Hughes) syndrome (APS). Lupus.

[117] Unterman A, Nolte JE, Boaz M, Abady M, Shoenfeld Y, Zandman-Goddard G (2011). Neuropsychiatric syndromes in systemic lupus erythematosus: a meta-analysis. Semin Arthritis Rheum.

[118] Zis P, Julian T, Hadjivassiliou M (2018) Headache Associated with Coeliac Disease: a systematic review and Meta-analysis. Nutrients 10(10). 10.3390/nu10101445 10.3390/nu10101445 PMC6213149 30301194

[119] Mortimer MJ, Kay J, Gawkrodger DJ, Jaron A, Barker DC (1993). The prevalence of headache and migraine in atopic children: an epidemiological study in general practice. Headache.

[120] Ku M, Silverman B, Prifti N, Ying W, Persaud Y, Schneider A (2006). Prevalence of migraine headaches in patients with allergic rhinitis. Ann Allergy Asthma Immunol.

[121] Ozge A, Ozge C, Oztürk C, Kaleagasi H, Ozcan M, Yalçinkaya DE, Ozveren N, Yalçin F (2006). The relationship between migraine and atopic disorders-the contribution of pulmonary function tests and immunological screening. Cephalalgia.

[122] Han JH, Lee HJ, Yook HJ, Han K, Lee JH, Park YM (2023). Atopic disorders and their risks of migraine: a Nationwide Population-based Cohort Study. Allergy Asthma Immunol Res.

[123] Becker C, Brobert GP, Almqvist PM, Johansson S, Jick SS, Meier CR (2008). The risk of newly diagnosed asthma in migraineurs with or without previous triptan prescriptions. Headache.

[124] Kang LL, Chen PE, Tung TH, Chien CW (2021). Association between Asthma and Migraine: a systematic review and Meta-analysis of Observational studies. Front Allergy.

[125] Wang L, Deng ZR, Zu MD, Zhang J, Wang Y (2020). The Comorbid Relationship between Migraine and Asthma: a systematic review and Meta-analysis of Population-Based studies. Front Med (Lausanne).

[126] Martelletti P, Sutherland J, Anastasi E, Di Mario U, Giacovazzo M (1989). Evidence for an immune-mediated mechanism in food-induced migraine from a study on activated T-cells, IgG4 subclass, anti-IgG antibodies and circulating immune complexes. Headache.

[127] Lassen LH, Haderslev PA, Jacobsen VB, Iversen HK, Sperling B, Olesen J (2002). CGRP may play a causative role in migraine. Cephalalgia.

[128] Goadsby PJ, Edvinsson L, Ekman R (1990). Vasoactive peptide release in the extracerebral circulation of humans during migraine headache. Ann Neurol.

[129] Cernuda-Morollón E, Larrosa D, Ramón C, Vega J, Martínez-Camblor P, Pascual J (2013). Interictal increase of CGRP levels in peripheral blood as a biomarker for chronic migraine. Neurology.

[130] Murray AM, Stern JI, Robertson CE, Chiang CC (2022). Real-world patient experience of CGRP-Targeting therapy for migraine: a Narrative Review. Curr Pain Headache Rep.

[131] Carucci JA, Ignatius R, Wei Y, Cypess AM, Schaer DA, Pope M, Steinman RM, Mojsov S (2000). Calcitonin gene-related peptide decreases expression of HLA-DR and CD86 by human dendritic cells and dampens dendritic cell-driven T cell-proliferative responses via the type I calcitonin gene-related peptide receptor. J Immunol.

[132] Voedisch S, Rochlitzer S, Veres TZ, Spies E, Braun A (2012). Neuropeptides control the dynamic behavior of airway mucosal dendritic cells. PLoS ONE.

[133] Mach DB, Rogers SD, Sabino MC, Luger NM, Schwei MJ, Pomonis JD, Keyser CP, Clohisy DR, Adams DJ, O’Leary P, Mantyh PW (2002). Origins of skeletal pain: sensory and sympathetic innervation of the mouse femur. Neuroscience.

[134] Li Y, Wang L, Gao Z, Zhou J, Xie S, Li G, Hou C, Wang Z, Lv Z, Wang R, Han G (2023). Neuropeptide CGRP promotes Immune Homeostasis of bacterial meningitis by inducing MHC-II ubiquitination. J Infect Dis.

[135] Duan JX, Zhou Y, Zhou AY, Guan XX, Liu T, Yang HH, Xie H, Chen P (2017). Calcitonin gene-related peptide exerts anti-inflammatory property through regulating murine macrophages polarization in vitro. Mol Immunol.

[136] Altmayr F, Jusek G, Holzmann B (2010). The neuropeptide calcitonin gene-related peptide causes repression of tumor necrosis factor-alpha transcription and suppression of ATF-2 promoter recruitment in toll-like receptor-stimulated dendritic cells. J Biol Chem.

[137] Monneret G, Arpin M, Venet F, Maghni K, Debard AL, Pachot A, Lepape A, Bienvenu J (2003). Calcitonin gene related peptide and N-procalcitonin modulate CD11b upregulation in lipopolysaccharide activated monocytes and neutrophils. Intensive Care Med.

[138] McGillis JP, Rangnekar V, Ciallella JR (1995). A role for calcitonin gene related peptide (CGRP) in the regulation of early B lymphocyte differentiation. Can J Physiol Pharmacol.

[139] Schlomer JJ, Storey BB, Ciornei RT, McGillis JP (2007). Calcitonin gene-related peptide inhibits early B cell development in vivo. J Leukoc Biol.

[140] Boudard F, Bastide M (1991). Inhibition of mouse T-cell proliferation by CGRP and VIP: effects of these neuropeptides on IL-2 production and cAMP synthesis. J Neurosci Res.

[141] Levite M (2000). Nerve-driven immunity. The direct effects of neurotransmitters on T-cell function. Ann N Y Acad Sci.

[142] Levite M (1998). Neuropeptides, by direct interaction with T cells, induce cytokine secretion and break the commitment to a distinct T helper phenotype. Proc Natl Acad Sci U S A.

[143] Mikami N, Matsushita H, Kato T, Kawasaki R, Sawazaki T, Kishimoto T, Ogitani Y, Watanabe K, Miyagi Y, Sueda K, Fukada S, Yamamoto H, Tsujikawa K (2011). Calcitonin gene-related peptide is an important regulator of cutaneous immunity: effect on dendritic cell and T cell functions. J Immunol.

[144] Feng G, Xu X, Wang Q, Liu Z, Li Z, Liu G (2010). The protective effects of calcitonin gene-related peptide on gastric mucosa injury after cerebral ischemia reperfusion in rats. Regul Pept.

[145] Tuka B, Helyes Z, Markovics A, Bagoly T, Szolcsányi J, Szabó N, Tóth E, Kincses ZT, Vécsei L, Tajti J (2013). Alterations in PACAP-38-like immunoreactivity in the plasma during ictal and interictal periods of migraine patients. Cephalalgia.

[146] Zagami AS, Edvinsson L, Goadsby PJ (2014). Pituitary adenylate cyclase activating polypeptide and migraine. Ann Clin Transl Neurol.

[147] Han X, Dong Z, Hou L, Wan D, Chen M, Tang W, Yu S (2015). Interictal plasma pituitary adenylate cyclase-activating polypeptide levels are decreased in migraineurs but remain unchanged in patients with tension-type headache. Clin Chim Acta.

[148] Hanci F, Kilinc YB, Kilinc E, Turay S, Dilek M, Kabakus N (2021). Plasma levels of vasoactive neuropeptides in pediatric patients with migraine during attack and attack-free periods. Cephalalgia.

[149] Amin FM, Hougaard A, Schytz HW, Asghar MS, Lundholm E, Parvaiz AI, de Koning PJ, Andersen MR, Larsson HB, Fahrenkrug J, Olesen J, Ashina M (2014). Investigation of the pathophysiological mechanisms of migraine attacks induced by pituitary adenylate cyclase-activating polypeptide-38. Brain.

[150] Delgado M, Munoz-Elias EJ, Gomariz RP, Ganea D (1999). VIP and PACAP inhibit IL-12 production in LPS-stimulated macrophages. Subsequent effect on IFNgamma synthesis by T cells. J Neuroimmunol.

[151] Delgado M, Pozo D, Martinez C, Leceta J, Calvo JR, Ganea D, Gomariz RP (1999). Vasoactive intestinal peptide and pituitary adenylate cyclase-activating polypeptide inhibit endotoxin-induced TNF-alpha production by macrophages: in vitro and in vivo studies. J Immunol.

[152] Delgado M, Munoz-Elias EJ, Gomariz RP, Ganea D (1999). Vasoactive intestinal peptide and pituitary adenylate cyclase-activating polypeptide enhance IL-10 production by murine macrophages: in vitro and in vivo studies. J Immunol.

[153] Martínez C, Delgado M, Pozo D, Leceta J, Calvo JR, Ganea D, Gomariz RP (1998). Vasoactive intestinal peptide and pituitary adenylate cyclase-activating polypeptide modulate endotoxin-induced IL-6 production by murine peritoneal macrophages. J Leukoc Biol.

[154] Delgado M, Leceta J, Gomariz RP, Ganea D (1999). Vasoactive intestinal peptide and pituitary adenylate cyclase-activating polypeptide stimulate the induction of Th2 responses by up-regulating B7.2 expression. J Immunol.

[155] Khan S, Amin FM, Fliedner FP, Christensen CE, Tolnai D, Younis S, Olinger ACR, Birgens H, Daldrup-Link H, Kjær A, Larsson HBW, Lindberg U, Ashina M (2019). Investigating macrophage-mediated inflammation in migraine using ultrasmall superparamagnetic iron oxide-enhanced 3T magnetic resonance imaging. Cephalalgia.

[156] Kim YJ, Granstein RD (2021). Roles of calcitonin gene-related peptide in the skin, and other physiological and pathophysiological functions. Brain Behav Immun Health.

[157] Kulka M, Sheen CH, Tancowny BP, Grammer LC, Schleimer RP (2008). Neuropeptides activate human mast cell degranulation and chemokine production. Immunology.

[158] Russell FA, King R, Smillie SJ, Kodji X, Brain SD (2014). Calcitonin gene-related peptide: physiology and pathophysiology. Physiol Rev.

[159] Liebman HA (2016) Immune modulation for autoimmune disorders: evolution of therapeutics. Semin Hematol 53 Suppl 1S23–26. 10.1053/j.seminhematol.2016.04.008 10.1053/j.seminhematol.2016.04.008 27312159

