# Supplementary material for: Altered immunity in migraine: a comprehensive scoping review
Source: J Headache Pain. 2024 Jun 7;25(1):95. doi: 10.1186/s10194-024-01800-8 (PMC11157828; doi:10.1186/s10194-024-01800-8)
Supplement: Supplementary file 1 — Supplementary Material 1 [file 10194_2024_1800_MOESM1_ESM.docx]

**Supplementary Materials**

**S1.** Forest plots of IL-1β levels in patients with migraine

**S2.** Forest plots of IL-6 levels in patients with migraine

**S3.** Forest plots of TNF-α levels in patients with migraine

**S4.** Forest plot of interictal IL-10 levels in patients with migraine, in comparison with controls

**S5.** Forest plots of interictal total ADP & leptin levels in patients with migraine, in comparison with controls

A.


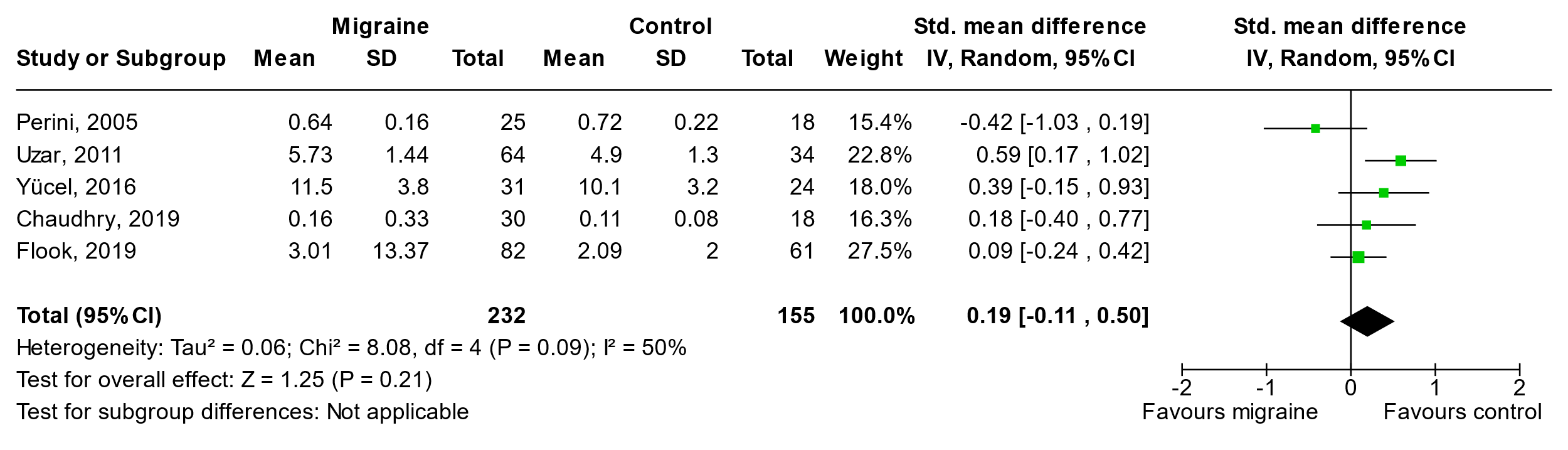


B.


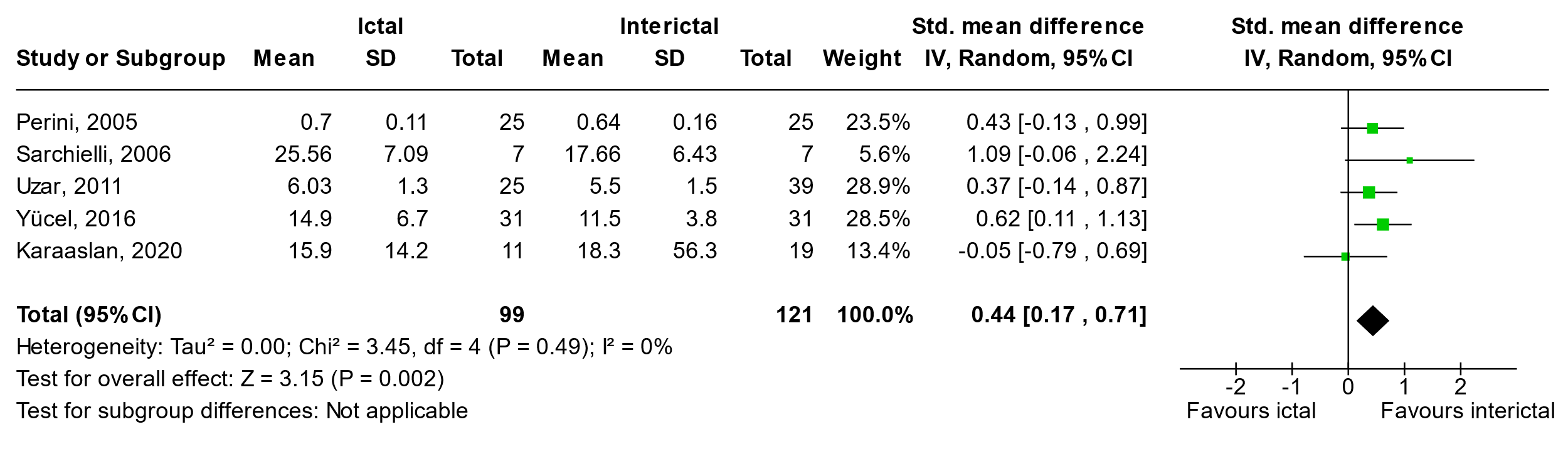


**S1.** Forest plots of IL-1β levels in patients with migraine A. Interictal IL-1β levels in comparison with controls B. Ictal IL-1β levels in comparison with interictal levels

A.


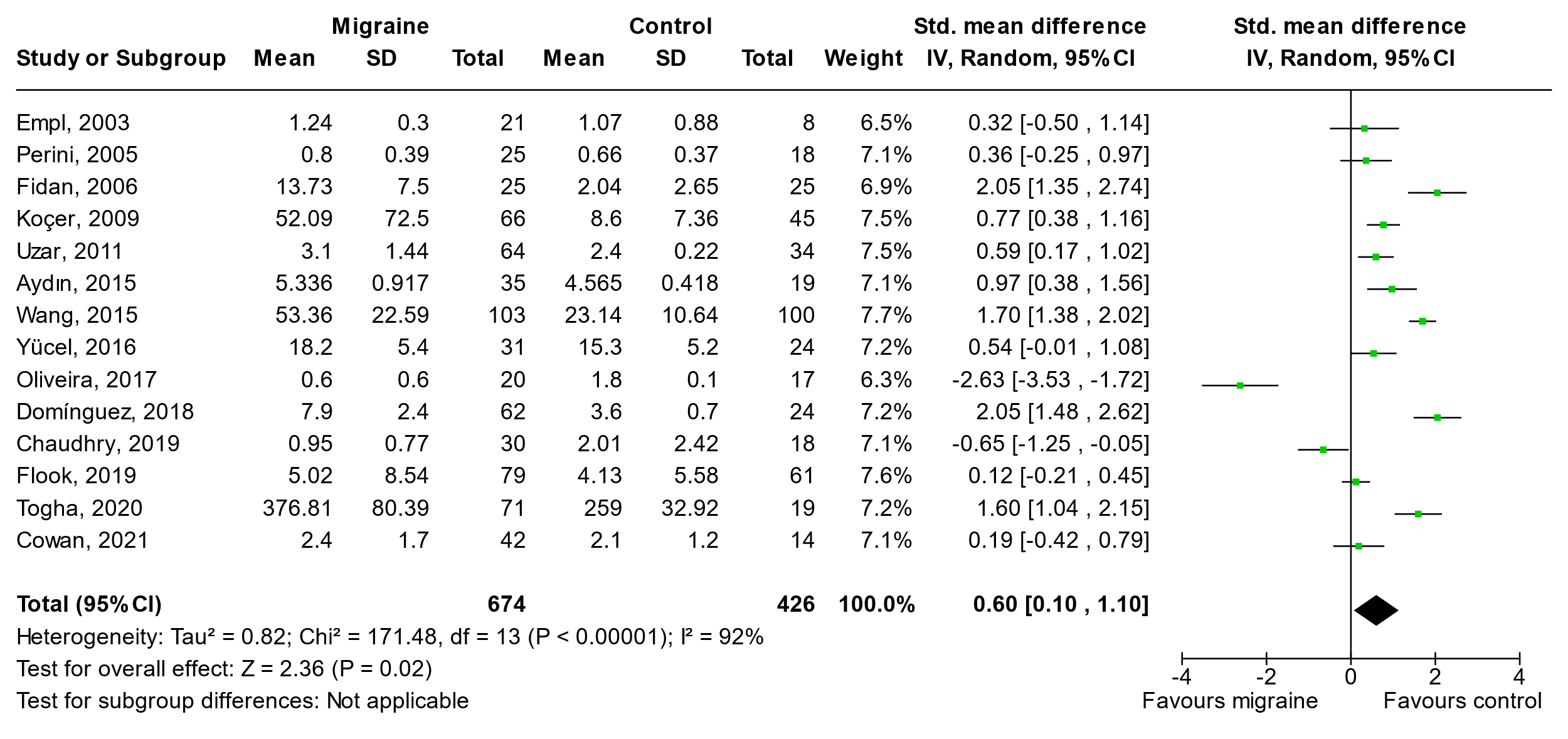


B.


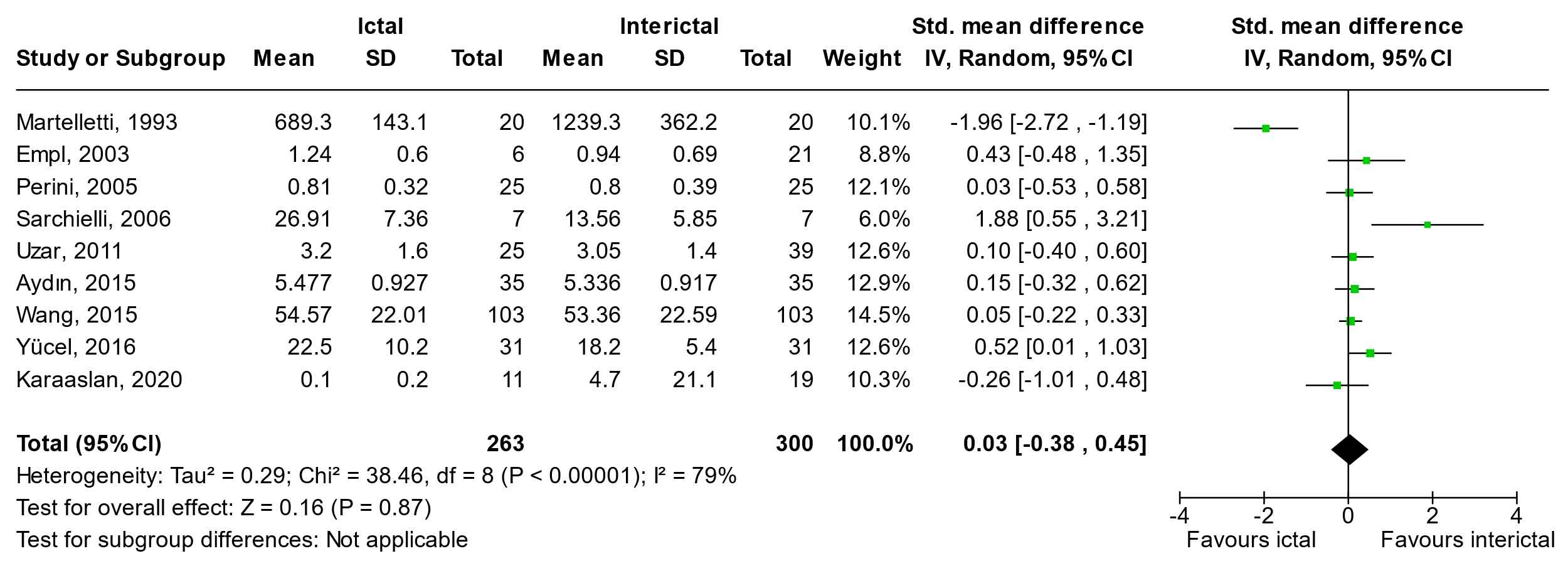


**S2.** Forest plots of IL-6 levels in patients with migraine A. Interictal IL-6 levels in comparison with controls B. Ictal IL-6 levels in comparison with interictal levels

A.


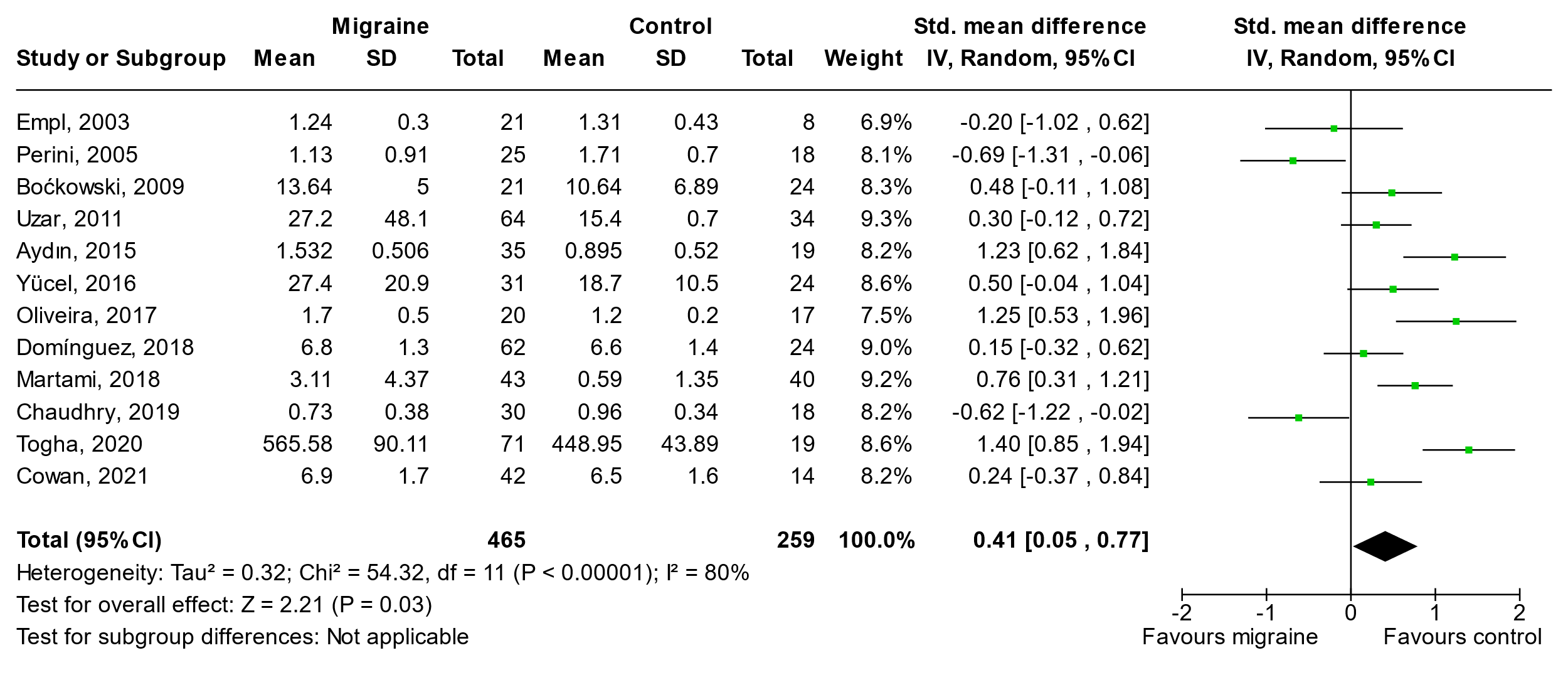


B.


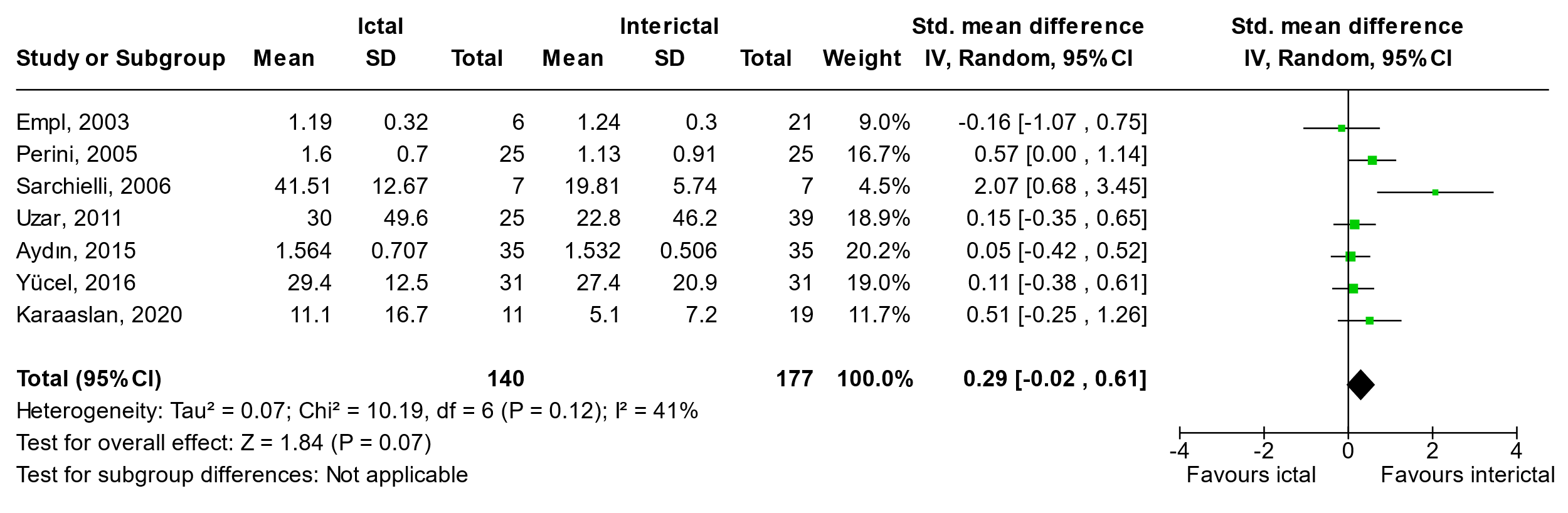


**S3.** Forest plots of TNF-α levels in patients with migraine A. Interictal TNF-α levels in comparison with controls B. Ictal TNF-α levels in comparison with interictal levels


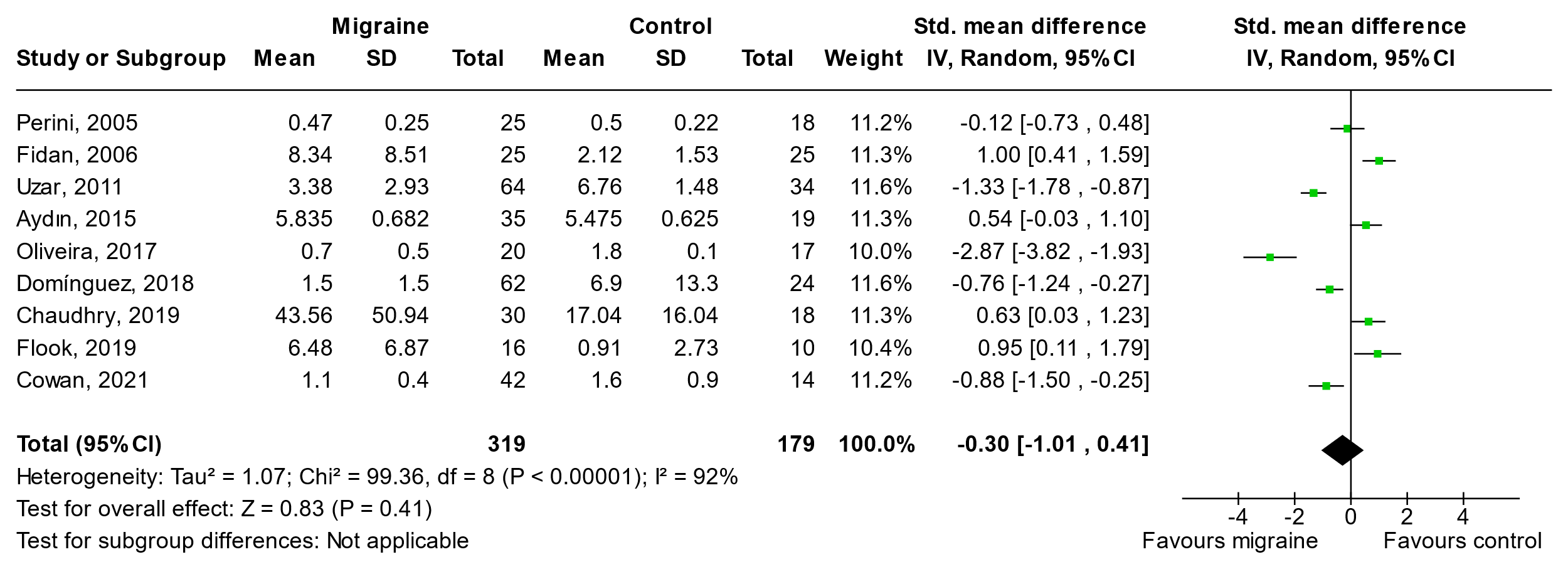


**S4.** Forest plot of interictal IL-10 levels in patients with migraine, in comparison with controls

A.


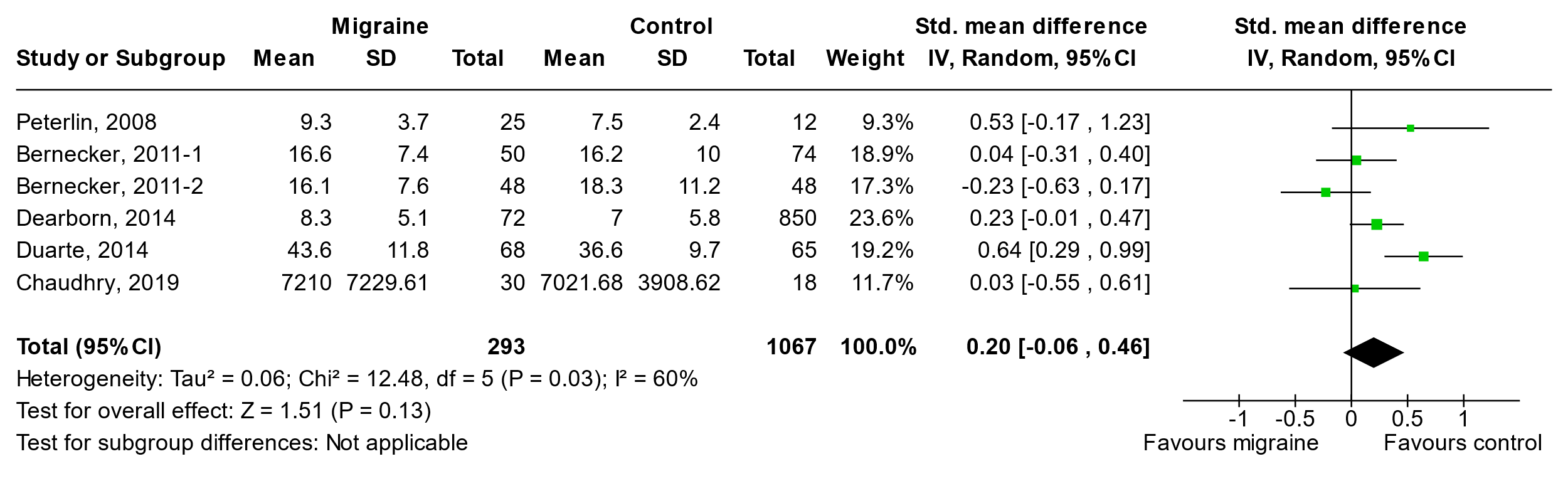


B.


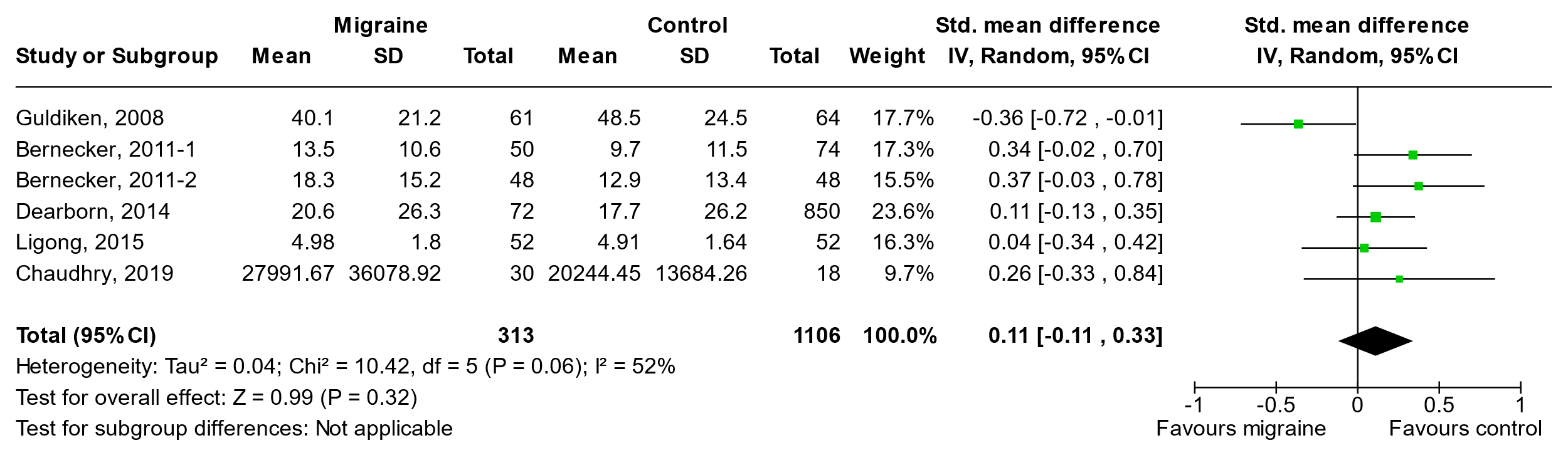


**S5.** Forest plots of interictal total ADP & leptin levels in patients with migraine, in comparison with controls A. Total ADP levels B. Leptin levels
